# A multiplexed parallel reaction monitoring assay to monitor bovine pregnancy-associated glycoproteins throughout pregnancy and after gestation

**DOI:** 10.1371/journal.pone.0271057

**Published:** 2022-09-23

**Authors:** Tony Krebs, Isabel Kilic, Lisa Neuenroth, Thierry Wasselin, Momchil Ninov, Jens Tetens, Christof Lenz

**Affiliations:** 1 Department of Animal Sciences, Georg-August-University, Goettingen, Lower Saxony, Germany; 2 Institute of Clinical Chemistry, University Medical Center Goettingen, Goettingen, Lower Saxony, Germany; 3 Bioanalytical Mass Spectrometry Group, Max Planck Institute for Multidisciplinary Sciences, Goettingen, Lower Saxony, Germany; 4 Center for Integrated Breeding Research, Georg-August-University, Goettingen, Lower Saxony, Germany; Bryant University, UNITED STATES

## Abstract

Bovine pregnancy-associated glycoproteins (**boPAGs**) are extensively glycosylated secretory proteins of trophoblast cells. Roughly 20 different boPAG members are known but their distribution patterns and degree of glycosylation during pregnancy are not well characterized. The objective of the present study was the development of a parallel reaction monitoring-based assay for the profiling of different boPAGs during pregnancy and after gestation. Furthermore, we investigated the effects of N-glycosylation on our analytical results. BoPAGs were purified from cotyledons of four different pregnancy stages. The assay detects 25 proteotypic peptides from 18 boPAGs in a single run. The highest abundances were found for boPAG 1 in both, glycosylated and deglycosylated samples. Strongest effects of glycosylation were detected during mid and late pregnancy as well as in afterbirth samples. Furthermore, we identified different boPAG-clusters based on the observed relative protein abundances between glycosylated and deglycosylated samples. A linkage between the impact of glycosylation and potential N-glycosylation sites or phylogenetic relation was not detected. In conclusion, the newly developed parallel reaction monitoring-based assay enables for the first time a comprehensive semi-quantitative profiling of 18 different boPAGs during pregnancy and post-partum on protein level, thereby investigating the influence of glycosylation. The results of this study provide new and important starting points to address further research on boPAGs to better understand their physiological role during pregnancy and for the development of new pregnancy detection tests.

## Introduction

Pregnancy-Associated Glycoproteins (**PAGs**) are expressed in trophoblast cells of the placenta of species within the Cetartiodactyla order and secrete in maternal blood and milk [1]. They belong to the aspartic proteinase family and are therefore related to pepsin [2, 3], cathepsin D, cathepsin E [4], chymosins and renin [4–6]. PAGs can be phylogenetically divided into an ancient group, which is predicted to have originated 87 million years ago, and a modern group, which is predicted to have arisen 52 million years ago [1, 7]. Most of the PAGs belong to the modern group, which can only be found in the Ruminantia with their synepitheliochorial placenta, and they are particular numerous in the *Bovidae* [1, 8–10].

To date, in cattle roughly 20 different PAGs and related paralogs are known, with largely varying temporal and spatial expression patterns during gestation [1, 9, 10]. Phylogenetic analysis discovered that six of those bovine PAGs (**boPAGs**) belong to the ancient group [7, 9, 11]. Initial studies suggest that ancient boPAGs are predominantly expressed in both, mono- and binucleate cells, whereas modern boPAGs are expressed only in binucleate cells [2, 9, 11]. A more recent study by Touzard et al. (2013) demonstrated that modern boPAGs are expressed in cotyledons and ancient boPAGs are expressed in the intercotyledonary chorion [12].

The ancient boPAGs are thought to be active aspartic proteinases, whereas modern boPAGs have lost their catalytic activity due to amino acid substitutions within the binding sites [3, 5, 11, 13]. The enzymatic activity for some members of the ancient group was experimentally confirmed [14]. In the modern boPAG group the binding function may be retained and enables this group to bind or interact with peptides or proteins without hydrolyzing them [3, 5, 11, 13].

An unusual feature of boPAGs is their high degree of glycosylation. The expected molecular mass of the protein core without posttranslational modifications and after the removal of the signal sequence is around 37 kDa [15, 16]. The mean molecular weight of boPAGs with attached N-glycans is around 67 kDa [12, 16–18]. Therefore, the degree of N-glycosylation seems to be the major factor in boPAG molecular mass [16]. The different boPAGs have up to six potential N-glycosylation sites [4, 12, 16]. A tetraantennary core-fucosylated structure with a bisecting N-acetylglucosamine (GlcNAc) with all antennae carrying a terminal Sd^a^-antigen (NeuAcα2–3[GalNAcβ1–4]Galβ1-4GlcNAc-) could be identified as the most abundant N-glycan in boPAGs expressed in binucleate cells [19]. This N-glycan structure shows a relatively high uniformity, which is indicative for a highly regulated glycosylation process in the binucleate cells and therefore suggests that the glycans might have specific functions during pregnancy [15, 19, 20]. Furthermore, the attached N-glycans and their change during the course of pregnancy seem to be an important factor for the boPAG-clearance from the maternal blood. The absence of the Sd^a^-antigen in the beginning (before day 30 of gestation) and to the end of pregnancy causes a higher serum half-life of boPAGs at these timepoints [19, 20]. This led to the assumption that the glycosylation process in cattle is under endocrine control. The change in estradiol concentration might be the main regulatory element in this process, but the exact mechanisms remain unclear [19]. Despite many years of research, the overall physiological role of boPAGs and the mechanism of their possible function are unknown. It has been hypothesized in the past that PAGs may act in protecting fetal or placental antigens from the maternal immune system, process growth factors, influence the secretion of progesterone, or facilitate adhesion (at implantation) and detachment (at birth) processes at the fetal maternal interface [1]. Therefore, their existence seems to be essential for a successful pregnancy outcome. To date, there are only a few studies that give insights into the protein level of different boPAGs [12, 16–19, 21]. However, these studies are often limited to few PAGs and a specific gestational period (e.g. mid pregnancy) and thus do not cover the entire pregnancy.

During the last years, new mass spectrometers and data acquisition schemes for the characterization and quantification of proteins have been developed [22–24]. In targeted MS methods, only a specific subset of analytes is measured in predefined *m/z* ranges and known retention time windows, e.g. a set of predefined tryptic peptides as surrogates for the proteins of interest [25, 26]. These methods have become the gold standard for large-scale quantification and verification of proteins, even when applied to complex biological samples [22, 23, 26, 27]. Parallel Reaction Monitoring (**PRM**) is a targeted method where all product ions of mass-selected peptides are monitored in parallel with one ion injection and full scan mass analysis [22, 24]. This method has several advantages. All potential product ions of a target peptide are available for peptide identification and there is no need of preselection of target transitions before analysis [22]. Furthermore, PRM has a high tolerance for co-isolated background peptides and can be multiplexed where the product ions of several target peptides are comingled and detected in a single-scan [22, 28].

The aim of the present study is the development and validation of a multiplexed PRM assay for boPAGs in order to allow for the first time a comprehensive semi-quantitative profiling of boPAGs during pregnancy and post-partum. Furthermore, we investigate glycosylated and deglycosylated samples to detect possible effects of protein modifications on the results obtained using our methods. The establishment of a new method for the characterization of boPAGs on protein level will enable future studies investigating the physiological role of boPAGs during pregnancy and developing new pregnancy detection tests.

## Materials and methods

The study is in accordance with the German legal and ethical requirements of appropriate animal procedures. Animals were not purposely euthanized for this study. Tissue samples were taken during the conventional slaughter process.

### Tissue collection and protein purification

The detailed workflow of tissue collection and subsequent protein purification is described in Krebs et al., 2021 [29]. In brief, cotyledon samples were collected from an abattoir and a local dairy farm. Based on the crown-rump length of the fetuses [30], they were divided into four pregnancy stages: 35–90 days of gestation (early pregnancy), 91–180 days of gestation (mid pregnancy), 181–240 days of gestation (late pregnancy) and afterbirth samples. Subsequently, protein extraction from cotyledonary tissue was performed according to the protocols of Zoli et al. (1991) [17] and Klisch et al. (2005) [16] with some modifications. Further protein purification was performed using a multistep fast protein liquid chromatography (**FPLC**). A scheme of the different FPLC steps can be found in S1 Fig in S2 File. The resulting boPAG-containing fractions were stored at -20°C until further analysis [29].

### Peptide-N-Glycosidase F (EC 3.5.1.52) treatment

Aliquots of protein samples from chromatography analysis were subjected to Peptide-N-Glycosidase F (**PNGaseF**) digestion to remove N-gylcans according to the manufacturer’s instructions (New England Biolabs, Germany). Therefore, 10 μg of protein extract were denatured for 10 min at 100°C in glycoprotein denaturing buffer 10x (New England Biolabs, Germany) and LiChrosolv H_2_O (Merck, Germany) (if necessary) in a 10 μl reaction volume. After cooling for 5 min on ice, the samples were centrifuged for 10 sec at full speed (13,300 rpm). Following this, 100 IU PNGaseF, 2 μl GlycoBuffer 2 (10x) (New England Biolabs, Germany), 2 μl 10% NP-40 (New England Biolabs, Germany) and 6 μl LiChrosolv H_2_O were added to the samples to reach a total reaction volume of 20 μl. After incubation for 1 h at 37°C, deglycosylated protein samples were used for in gel digestion followed by mass spectrometric analysis.

### Western blot analysis

Purified protein samples from homogenized cotyledonary tissue of all pregnancy stages were either deglycosylated with PNGase F or left glycosylated. All samples were diluted to a final concentration of 1 μg protein/lane in either deglycosylation buffer (deglycosylated samples) or with dd H_2_O (glycosylated samples). In addition, NuPAGE LDS sample buffer 4x (Thermo Fisher Scientific, USA) was added to all samples, followed by an incubation for 10 min at 70°C prior loading.

Gel electrophoresis was performed using NuPAGE 4–12% Bis-Tris 1.0 mm gradient gels (Thermo Fisher Scientific, USA). Gels were run at 80 V until the samples were migrated out of the wells. Afterwards the voltage was increased to 150 V to finish the run. Molecular weight standard (Protein Marker VI (10–245) prestained, Applichem, Germany) was run simultaneously. Gels were either stained with Coomassie Brilliant Blue or proteins were transferred onto nitrocellulose membrane (GE Healthcare, USA) in transfer buffer (25 mM Tris, 192 mM glycine diluted in 20% C_3_H_8_O) [31]. The transfer was performed by 80 V for 1.5 h at 4°C.

After transfer, the nitrocellulose membrane was briefly rinsed with ddH_2_O and stained with Ponceau S solution (0.1% Ponceau diluted in 5% CH_3_COOH) to check the protein transfer quality. Afterwards Ponceau stain was rinsed off with washes of Tris-buffered saline containing Tween 20 (TBST) (20 mM Tris, 136.5 mM NaCl diluted in 0.01% Tween 20; pH 7.4).

The membranes were blocked with 1% BSA in TBST at room temperature (**RT**) for 30 min, washed three times with TBST, and probed with different boPAG antisera at a concentration of 13 μg antibody/membrane. Polyclonal boPAG antisera were produced using seven boPAG-fractions of different pregnancy stages from chromatography. A detailed description of the polyclonal antibody production is given in Krebs et al. (2021) [29]. Afterwards, membranes were incubated overnight at 4°C while shaking. On the next day, the nitrocellulose membranes were washed three times with TBST and incubated with a fluorescence-labeled goat anti-rabbit IgG (1:50,000) (IRDye 680RD Goat anti-Rabbit IgG Secondary Antibody, LI-COR Biosciences, USA) for 1 h at room temperature in the dark. After three TBST washes, western blots were developed at 700 nm with Odyssey CLx Near-Infrared Fluorescence Imaging System (LI-COR Biosciences, USA).

### Mass spectrometric protein analysis–sample preparation

Acetonitrile and H_2_O used in these experiments were purchased from Merck, Germany in LiChrosolv hypergrade.

Both glycosylated samples from chromatography and deglycosylated samples from PNGaseF digestion were subjected to in-gel digestion with trypsin (EC 3.4.21.4). Therefore, all samples were diluted to a final concentration of 10 μg protein/lane in NuPAGE LDS sample buffer 4x, NuPAGE Sample Reducing Agent 10x (Thermo Fisher Scientific, USA) and H_2_O, followed by incubation for 10 min at 70°C prior to loading. Gel electrophoresis was performed on a NuPAGE 4–12% Bis-Tris 1.0 mm gradient gel in an 8x8 cm vertical unit (Bio-Rad, USA) and stained with Coomassie Brilliant Blue for visualization. For an initial shotgun analysis to determine sample purity and detect PRM peptide candidates, gels were run full distance and cut into 15 equidistant slices irrespective of staining. For the final PRM assays sample were run ~1 cm into the gel for purification purposes only and excised as a single band. Gel slices were washed 5 min at room temperature under shaking. Afterwards, the supernatant was discarded and 100% acetonitrile (Merck, Germany) added to the gel pieces. After incubation for 15 min at room temperature on a shaker (750 rpm), the supernatant was again discarded and the gel pieces were dried in a vacuum centrifuge (SpeedVac Savant SPD111V, Thermo Fisher Scientific, USA).

Reduction was performed by incubation with dithiothreitol (10 mM C_4_H_10_O_2_S_2_ in 100 mM NH_4_HCO_3_, 50 min, 56°C). Following centrifugation, the supernatant was removed and the gel pieces dried using 100% acetonitrile again. Alkylation was then achieved with iodoacetamide (55 mM C_2_H_4_INO in 100mM NH_4_HCO_3_, 20 min, RT, darkness). After removal of the supernatant, gel pieces were washed again with 100 mM NH_4_HCO_3_. Samples were dried using 100% acetonitrile again twice, and the gel pieces dried in a vacuum centrifuge. Endoprotease lysis was performed overnight at 37°C using modified porcine trypsin (Trypsin Gold, Promega, USA) in digestion buffer (50 mM NH_4_HCO_3_, 5 mM CaCl_2_) at an enzyme-to-protein ratio of 1:50.

Peptides were extracted from the gel by incubation with water (20 μl, 15 min, 37°C). 80 μl neat acetonitrile were added and further incubated 15 min at 37°C. The supernatants were transferred to fresh tubes, the gel pieces incubated again with 5% aqueous formic acid (65 μl, 15 min, 37°C), centrifuged, and incubated again after addition of 65 μl neat acetonitrile. Subsequently, the gel pieces were centrifuged and the supernatants pooled with supernatants from the first extraction. Resulting peptide solutions were dried in a vacuum centrifuge and stored at -20°C until further use.

Prior to MS analysis, peptide mixtures were reconstituted in loading buffer (1% acetonitrile, 0.1% CH_2_O_2_ in H_2_O) to a nominal concentration of 100 fmol/μl, and spiked with 100 fmol/μl *Escherichia coli* ß-galactosidase (EC 3.2.1.23) tryptic digest to avoid adsorption effects [32] and 100 fmol/μl of pepstatin A (Sigma-Aldrich, USA) to inhibit proteolytic activity of boPAGs [13, 14, 33] during analysis.

### Mass spectrometric protein analysis

For initial protein identification, samples were enriched on a self-packed reversed phase-C18 precolumn (0.15 mm ID x 20 mm, Reprosil-Pur120 C18-AQ 5 μm, Dr. A. Maisch HPLC GmbH, Germany) and separated on an analytical reversed phase-C18 column (0.075 mm ID x 200 mm, Reprosil-Pur 120 C18-AQ, 3 μm, Dr. A. Maisch HPLC GmbH, Germany) using a 30 min linear gradient of 5–35% acetonitrile/0.1% formic acid (v:v) at 300 nl/min). The eluent was analyzed on a Q Exactive hybrid quadrupole/orbitrap mass spectrometer (Thermo Fisher Scientific, Germany) equipped with a FlexIon nanoSpray source and operated under Excalibur 2.4 software using a data-dependent acquisition method. Each experimental cycle was of the following form: one full MS scan across the 350–1600 *m/z* range was acquired at a resolution setting of 70,000 full width at half maximum (**FWHM**), and AGC target of 1*10e^6^ and a maximum fill time of 60 ms. Up to the 12 most abundant peptide precursors of charge states 2 to 5 above a 2*10e^4^ intensity threshold were then sequentially isolated at 2.0 FWHM isolation width, fragmented with nitrogen at a normalized collision energy setting of 25%, and the resulting product ion spectra recorded at a resolution setting of 17,500 FWHM, and AGC target of 2*10e^5^ and a maximum fill time of 60 ms. Selected precursor *m/z* values were then excluded for the following 15 s. Two technical replicates per sample were acquired.

For PRM assays, samples were analyzed on a nanoflow chromatography system (Eksigent nanoLC425) hyphenated to a hybrid triple quadrupole-TOF mass spectrometer (TripleTOF 5600+) equipped with a Nanospray III ion source (Ionspray Voltage 2400 V, Interface Heater Temperature 150°C, Sheath Gas Setting 12) and controlled by Analyst TF 1.7.1 software build 1163 (all AB Sciex, Germany). Peptides dissolved in loading buffer were enriched on a micro pillar array trapping column (μPac 1 cm, 5 μm, PharmaFluidics, Belgium) and separated on an analytical micro pillar array column (μPac 50 cm, 2.5 μm, PharmaFluidics, Belgium) using a 30 min linear gradient of 5–35% acetonitrile/0.1% formic acid (v:v) at 300 nl/min.

Targeted LC/MS/MS analysis was performed using a Top12 parallel reaction monitoring acquisition consisting of a MS survey scan of *m/z* 350–1250 accumulated for 150 ms at a resolution of 30,000 FWHM, and up to 12 MS/MS scans of *m/z* 180–1600 accumulated for 150 ms at a resolution of 17,500 FWHM and a precursor isolation width of 0.7 FWHM, resulting in a total cycle time of 2.1 s. Precursors were chosen from a retention-time encoded list of peptide *m/z* values of interest, which were selected for MS/MS in a time window of RT±4 min above a threshold MS intensity of 125 cps. MS/MS activation was achieved by CID using nitrogen as a collision gas and the manufacturer’s default rolling collision energy settings. Two technical replicates per sample were acquired.

### PRM peptide selection and data analysis

Protein and peptide identification was achieved using MASCOT Software version 2.4 (Matrix Science Ltd, USA) [34] or MaxQuant Software version 1.5.7.4 (Max Planck Institute for Biochemistry, Germany) [35]. Proteins were identified against the UniProtKB bovine reference proteome (v2020.01) along with a set of common lab contaminants. Searches were performed with trypsin (excluding proline-proximal cleavage sites) as enzyme and iodoacetamide as cysteine blocking agent. Up to two missed tryptic cleavages were allowed for, and methionine oxidation and protein N-terminal acetylation variable modifications. Instrument type ‘Orbitrap’ was selected to adjust for MS acquisition specifics. MASCOT searches were performed using precursor mass tolerances of 15 ppm and a fragment mass tolerance of 0.1 Da. MaxQuant searches used an internal pre-calibration for a final search with tolerances of 4.5 ppm (MS) and 20 ppm (MS/MS), respectively. Protein and peptide results lists were thresholded at False Discovery Rates (**FDR**) of 0.01, respectively, using a forward-and-reverse decoy database approach. Afterwards, protein identification results were imported into Scaffold version 5.0.0 (Proteome Software, USA) [36] for analysis of sequence coverage of different boPAGs.

PRM data were analyzed using Skyline version 20.1.0.76 (University of Washington, USA) [37]. In Skyline, all peaks were automatically integrated and the 6 most intense transitions (y and b ions) for each precursor were selected. Furthermore, all peaks were manually inspected and the peak boundaries were adjusted to avoid interferences and to confirm correct detection. Afterwards the Total Area Fragment data from each peptide was exported from Skyline and further analyzed using R 3.6.1 (R Development Core Team, Austria).

PRM for qualitative measurements of boPAGs in the protein digests were performed targeting at least one prototypic peptide for each boPAG (Table 1). Selected peptides were verified to be unique to the protein of interest by an online BLAST analysis (Program: NCBI BLASTP, database: NCBI Protein Reference Sequences database, Organism: Bos Taurus (taxid:9913), 2020/06/22). Peptides containing potential missed cleavage sites, methionine or cysteine were excluded; doubly charged precursor ions were favored. Furthermore, the selected peptides must have a length between 8 and 25 amino acids and *m/z* values between 450 and 800.

**Table 1:** List of proteins and proteotypic peptides.

PAG	Peptide Sequence	Precursor Charge	Average Measured RT (sec)	Precursor *m/z*	Mean CV (%)
PAG 1 (NP_776836.1)	R.VSSSTETWYLGDVFLR.L	3	42.48	620.64	8.72
PAG 2 (NP_788787.1)	K.TFNPQNSSSFR.E	2	42.13	642.80	11.75
	R.NYLDTAYVGNITIGTPPQEFR.V	3	38.34	790.39	16.07
PAG 3 (NP_001291497.1)	K.VSSSTETWILGDVFLR.V	3	40.71	603.98	16.58
PAG 4 (NP_788788.1)	K.ALVDTGSSDIVGPSTLVNNIWK.L	3	40.32	762.73	20.32
PAG 5 (NP_788789.1)	R.HLESSTSGLTQK.T	2	40.96	644.33	13.11
	K.ENTVSTSTETWILGDVFLR.L	3	42.29	723.36	10.86
PAG 6 (NP_788790.1)	K.GIPFDGILGLSYPNK.T	3	41.11	530.95	10.33
PAG 7 (NP_001103448.1)	R.HLQSSTFRPTNK.T	3	33.16	472.58	19.30
	K.WVPLIQAVDWSVHVDR.I	3	43.35	640.67	8.86
PAG 8 (NP_788792.2)	K.NLGTSETWILGDVFLR.L	3	41.72	607.65	
PAG 9 (NP_788793.1)	K.GELNWIPLIEAGEWR.V	3	32.91	594.97	11.46
PAG 10 (NP_788794.2)	R.IGNLVSVAQPFGLSLK.E	3	42.04	548.32	18.38
	R.TITGANPIFDNLWK.Q	2	42.50	795.41	12.34
PAG 11 (NP_788796.1)	K.QQGAISEPIFAFYLSTR.K	3	42.13	643.33	13.69
	R.VVFDTGSSDLWVPSIK.C	3	40.46	583.97	11.24
PAG 14 (XP_002699292.1)	R.NISFSGAIPIFYK.L	2	30.58	728.89	11.52
PAG 15 (NP_788797.1)	R.LSQISFHGSNLTIHPLR.N	3	43.36	640.68	27.29
PAG16 (NP_788798.1)	R.HFQSSTFRPTTK.T	2	41.72	718.86	4.47
	K.NQGAISDPIFAFYLSK.D	3	42.43	590.97	4.54
PAG 17 (NP_788800.1)	K.EHTYSLSQISSR.G	3	29.57	469.90	7.96
PAG 18 (NP_788799.1)	K.LSFSGAIPIFDNLR.N	3	23.35	517.28	10.12
PAG 20 (NP_788802.1)	R.FDGVLGLNYPNISFSK.A	3	42.43	590.97	9.14
	R.STEFWILGEAFLR.L	3	40.16	523.60	9.62
PAG 21 (NP_788803.1)	K.NEGAISEPIFAFYLSK.K	3	41.22	595.97	12.99

### Statistical analysis

All experimental results were statistically analyzed with R 3.6.1 (R Development Core Team, Austria). Peptides with a high coefficient of variation (CV) of their measured peak area between two technical replicates (CV > 30%) were not considered for further analysis. The relative protein abundances (expressed in percentage) of each boPAG (glycosylated and deglycosylated) in each pregnancy stage were calculated by the ratio of its peptide peak area to the total peptide peak area from all boPAGs of each pregnancy stage. This is a common method, which is also described in the literature [38, 39]. Relative protein levels and the influence of glycosylation during pregnancy were examined using a one-way ANOVA. In order to evaluate the differences within the classes of boPAGs, a post hoc test with Bonferroni correction was performed. The Bonferroni correction was applied to correct for the number of comparisons resulting from multiple testing. The results were considered significant at P < 0.05.

The shotgun MS proteomics data have been deposited to the ProteomeXchange Consortium via the PRIDE [40] partner repository with the dataset identifier PXD027383. The PRM raw data have also been deposited to the ProteomeXchange Consortium via the PRIDE [40] partner repository with the dataset identifier PXD034108.

## Results

In this study we developed a multiplexed PRM assay for boPAGs with the aim to provide an overview of the relative abundances of different boPAGs on protein level in the course of pregnancy and after parturition.

BoPAGs were purified from cotyledons from different gestation stages by FPLC. A detailed description of the purification process can be found in Krebs et al. (2021) [29]. A scheme of the different FPLC steps is shown in S1 Fig in S2 File. Overall, we analyzed six different purified poolsamples from four different gestation stages out of 16 different pregnancies (early pregnancy poolsample was purified from cotyledonary tissues of five male and five female fetuses, mid pregnancy, late pregnancy, and afterbirth poolsamples were purified from cotyledonary tissues of one male and one female fetus each) by mass spectrometry. The early pregnancy poolsample consisted of cotyledonary tissues of ten pregnant cows in order to obtain sufficient protein amounts. At this point, we would also like to mention that our workflow for developing the presented PRM assay makes use of multi-stage chromatographic separation upfront to the actual proteomic analysis (see Materials and Methods). The required effort technically precludes the use of high n sampling numbers.

**Fig 1:**
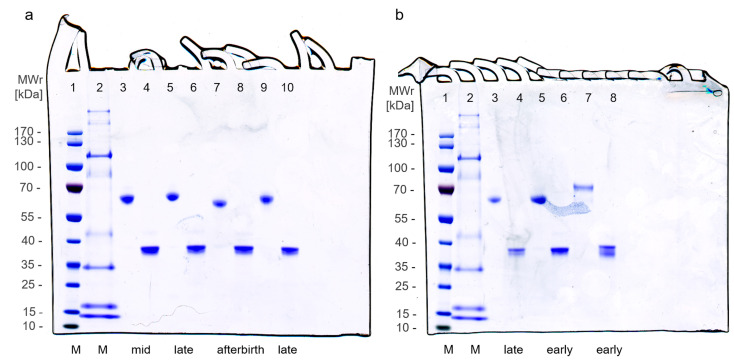
Gel images of seven different purified bovine pregnancy-associated glycoproteins (boPAG) samples from four different pregnancy stages. The protein samples (1 μg/lane) were either enzymatically deglycosylated with Peptide-N-Glycosidase F (PNGase F) (lane 4, 6, 8, 10) or left untreated (lane 3, 5, 7, 9). Molecular weights of the marker (M)-bands (lane 1, 2) are indicated on the left (kDa). Early pregnancy samples of lane 7 (b) and lane 8 (b) were not analyzed by mass spectrometry.

Upon gel electrophoresis, boPAG-fractions isolated from the cotyledonary tissue gave one major band at around 67 kDa in the native form and at approximately 37 kDa after deglycosylation (Fig 1). These apparent molecular weights are consistent with earlier data [1, 3, 16–18] including the mass shift after deglycosylation [12, 16, 20].

For an initial overview of the purity and composition of the different protein fractions, the glycosylated and deglycosylated samples were subjected to in-gel trypsin digestion followed by shotgun-MS analysis. From the data, a total of 9 different boPAGs (boPAG 1, boPAG 3, boPAG 4, boPAG 6, boPAG 7, boPAG 10, boPAG 16, boPAG 20 and boPAG 21) could be identified in the samples of the respective gestation stages (S1-S12 Tables in S3 File). We used the untargeted peptide identification results to construct a first list of candidate peptides for a targeted PRM assay. Due to the known limitations of shotgun mass spectrometry analyses to detect especially low abundant proteins [22, 41–43], we then extended the list to all 21 known boPAGs using predicted tryptic peptides. Protein sequences from UniprotKB and NCBI databases were theoretically digested with trypsin, and the resulting predicted peptide sequences were selected for suitability based on empirical criteria described in the Material and Methods section of this article. Due to the high degree of sequence homology among boPAGs, the resulting list of potentially ‘proteotypic’ unique peptide proxies was limited (S13 Table in S3 File).

We tested our PRM method based on this inclusion list of measured and predicted peptides on pooled reference samples that contained equal volumes of each of the boPAG samples from different gestational stages. After careful manual evaluation of peptide detectability, a final inclusion list of peptides (Table 1) was generated. Note that for boPAGs 12, 13 and 19, no suitable peptides could be found that fulfilled the criteria. As consequence, they were not considered in the further analysis. Based on the selected peptides and optimized parameters, we analyzed all samples of the respective pregnancy stages according the protocol described in the Material and Methods section. In summary, the final PRM analysis monitors 25 peptides (with their 6 most intense transitions) from 18 boPAGs using a scheduled inclusion list. A figure with the mapping of the chosen peptides onto the 18 sequences can be found in the S1 File.

### PAG profiles in glycosylated samples during gestation and post-partum

**Fig 2:**
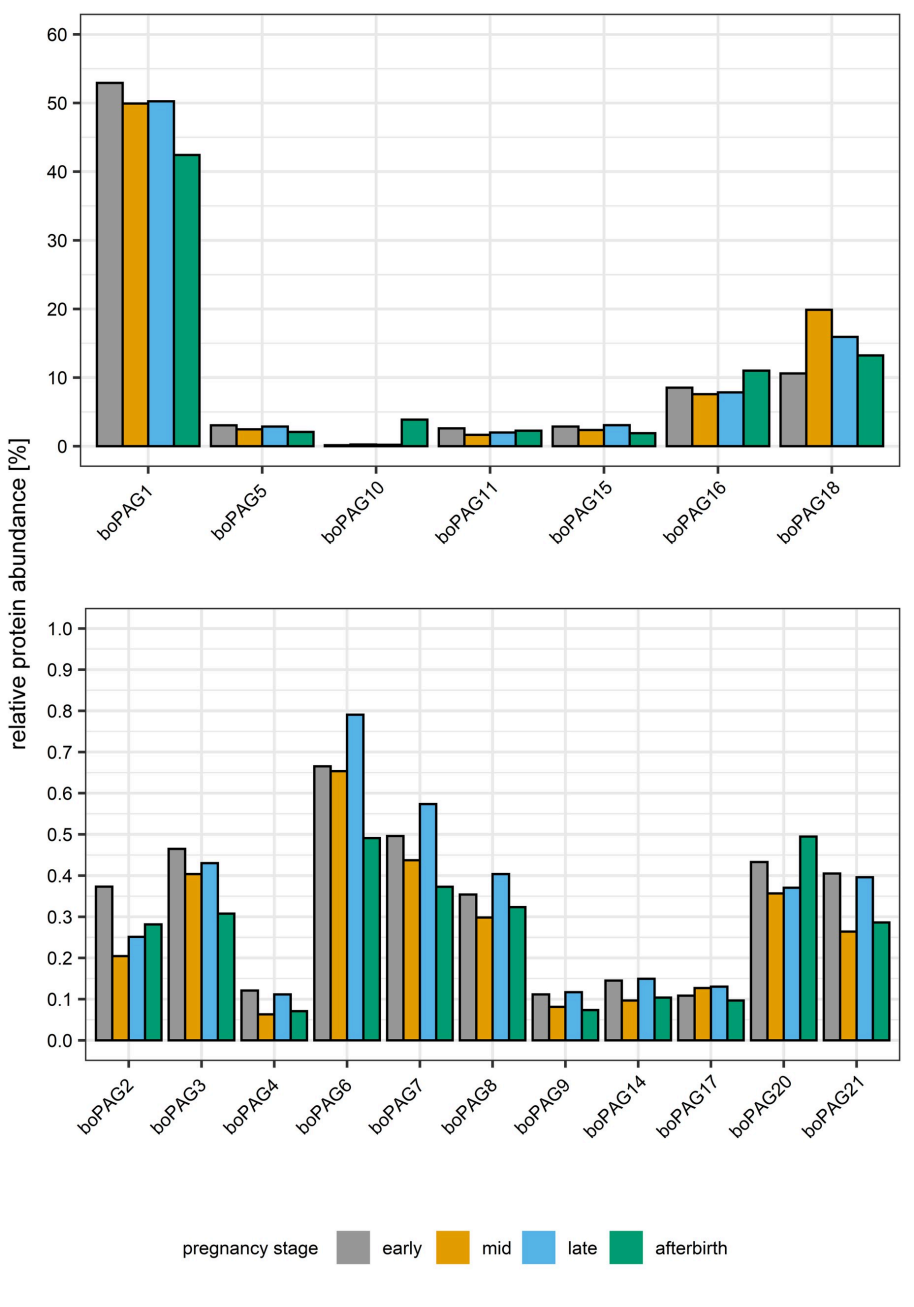
Visualization of relative protein abundances measured by Parallel Reaction Monitoring (PRM) mass spectrometry in glycosylated samples during pregnancy and post-partum.

For the evaluation of the relative abundances from the 18 different boPAGs during gestation and post-partum, we analyzed the six different purified samples from the different pregnancy stages using the developed PRM assay. The values of the late pregnancy samples were averaged. These data were used to calculate the relative abundance of each boPAG in the respective pregnancy stage. The mean relative abundances of boPAGs in the course of pregnancy and post-partum are shown in Fig 2.

Analysis of the data with ANOVA indicated a significant effect of boPAG-type on relative abundances (P < 0.001). BoPAG 1 was the most abundant boPAG in all pregnancy stages and showed a significant difference (P < 0.001) in mean relative abundance compared to the other investigated boPAGs. Furthermore, boPAG 18 showed significantly higher relative abundances (P < 0.001) in comparison to boPAG 2, boPAG 3, boPAG 4, boPAG 5, boPAG 6, boPAG 7, boPAG 8, boPAG 9, boPAG 10, boPAG 11, boPAG 14, boPAG 15, boPAG 17, boPAG 20, boPAG 21 and boPAG 16 (P = 0.005). BoPAG 16 had a higher level of mean relative abundance (P < 0.001) compared to boPAG 2, boPAG 3, boPAG 4, boPAG 5, boPAG 6, boPAG 7, boPAG 8, boPAG 9, boPAG 10, boPAG 11, boPAG 14, boPAG 17, boPAG 20, boPAG 21 and boPAG 15 (P = 0.005) throughout the period of gestation studied. The additionally observed differences between boPAGs did not reach statistical significance. Nevertheless, we detected distribution patterns in regard to boPAG concentrations at different stages in the course of pregnancy. The first group (boPAG 1; boPAG 5; boPAG 15) showed nearly equal relative mean abundances in early, mid and late pregnancy and decreased levels of protein post-partum. A second group (boPAG 2; boPAG 3; boPAG 4; boPAG 6; boPAG 7; boPAG 8; boPAG 9; boPAG 14; boPAG 21) displayed highest levels of relative mean abundances at early and late pregnancy stage in comparison to the levels observed at mid pregnancy and after gestation. BoPAG 17 and boPAG 18 exhibited a profile with highest levels during mid and late pregnancy. The last set comprised boPAG 10, boPAG 16 and boPAG 20. Within this group, we detected highest levels of relative abundance in the afterbirth sample.

### PAG profiles in deglycosylated samples during gestation and post-partum

The possible effects of glycosylation on mass spectrometry analyses are well known. Their complexity and associated physical properties can lead to an overall poorer detection of glycosylated proteins or peptides [42, 44–47]. Since boPAGs are highly glycosylated [15, 16, 19, 20], we decided to examine the effect of N-glycosylation on the outcome of our analysis.

**Fig 3:**
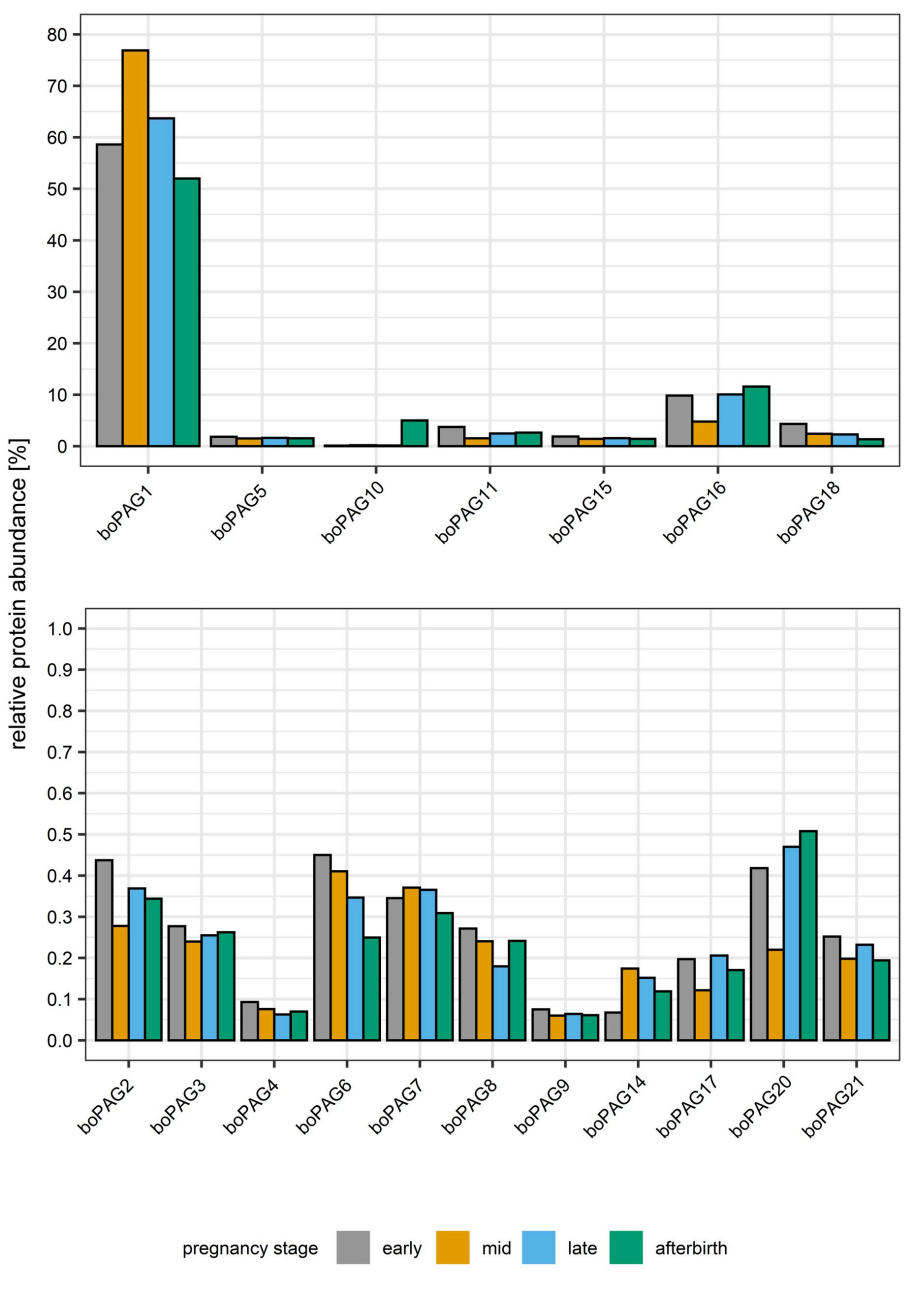
Visualization of relative protein abundances measured by Parallel Reaction Monitoring (PRM) mass spectrometry in deglycosylated samples during pregnancy and post-partum.

We subjected six samples purified from cotyledonary tissues at different pregnancy stages to enzymatic deglycosylation. The success of the Peptide-N-Glycosidase F (PNGase F) treatment was verified by gel electrophoresis (Fig 1). The observed differences in apparent molecular weight between glycosylated and deglycosylated samples already indicate the major effect of N-glycosylation on this this group of proteins. Changes in the mean relative abundances of deglycosylated boPAGs in the course of pregnancy and post-partum as detected by PRM mass spectrometry are shown in Fig 3.

ANOVA pointed out significant differences in the relative abundance of boPAGs (P < 0.001). Again, boPAG 1 was the most abundant boPAG at all pregnancy stages and showed significant differences (P < 0.001) in mean relative abundance compared to the other investigated boPAGs. Furthermore, boPAG 16 showed significantly higher relative abundances in comparison to boPAG 2 (P < 0.001), boPAG 3 (P = 0.009), boPAG 4 (P = 0.006), boPAG 5 (P = 0.005), boPAG 6 (P = 0.01), boPAG 7 (P < 0.001), boPAG 8 (P = 0.008), boPAG 9 (P = 0.006), boPAG 10 (P = 0.003), boPAG 11 (P = 0.04), boPAG 14 (P = 0.007), boPAG 17 (P = 0.007), boPAG 20, (P < 0.001) and boPAG 21 (P = 0.008). The observed differences between other boPAGs did not reach statistical significance.

Nevertheless, mean relative boPAG levels could be assigned to different groups of nearly identical distribution patterns. BoPAG 1, boPAG 7 and boPAG 14 showed highest relative levels in mid pregnancy compared to the other pregnancy stages. Another group (boPAG 5; boPAG 9; boPAG 15) showed nearly equal relative mean abundances during gestation and after parturition. BoPAG 2, boPAG 3, boPAG 4, boPAG 17 and boPAG 21 had highest levels of mean relative abundances during early and late pregnancy compared to their levels during mid pregnancy and post-partum. Some boPAGs, such as boPAG 4, boPAG 6, boPAG 8 and boPAG 18 showed decline in relative abundances in the course of pregnancy with highest levels reached at early pregnancy stage. Two boPAGs (boPAG 4 and boPAG 8) had increased levels in the post-partum sample. The last set comprised boPAG 10, boPAG 16 and boPAG 20 which exhibited highest levels of relative mean abundances in the afterbirth sample.

### Comparison between glycosylated and deglycosylated samples

**Fig 4:**
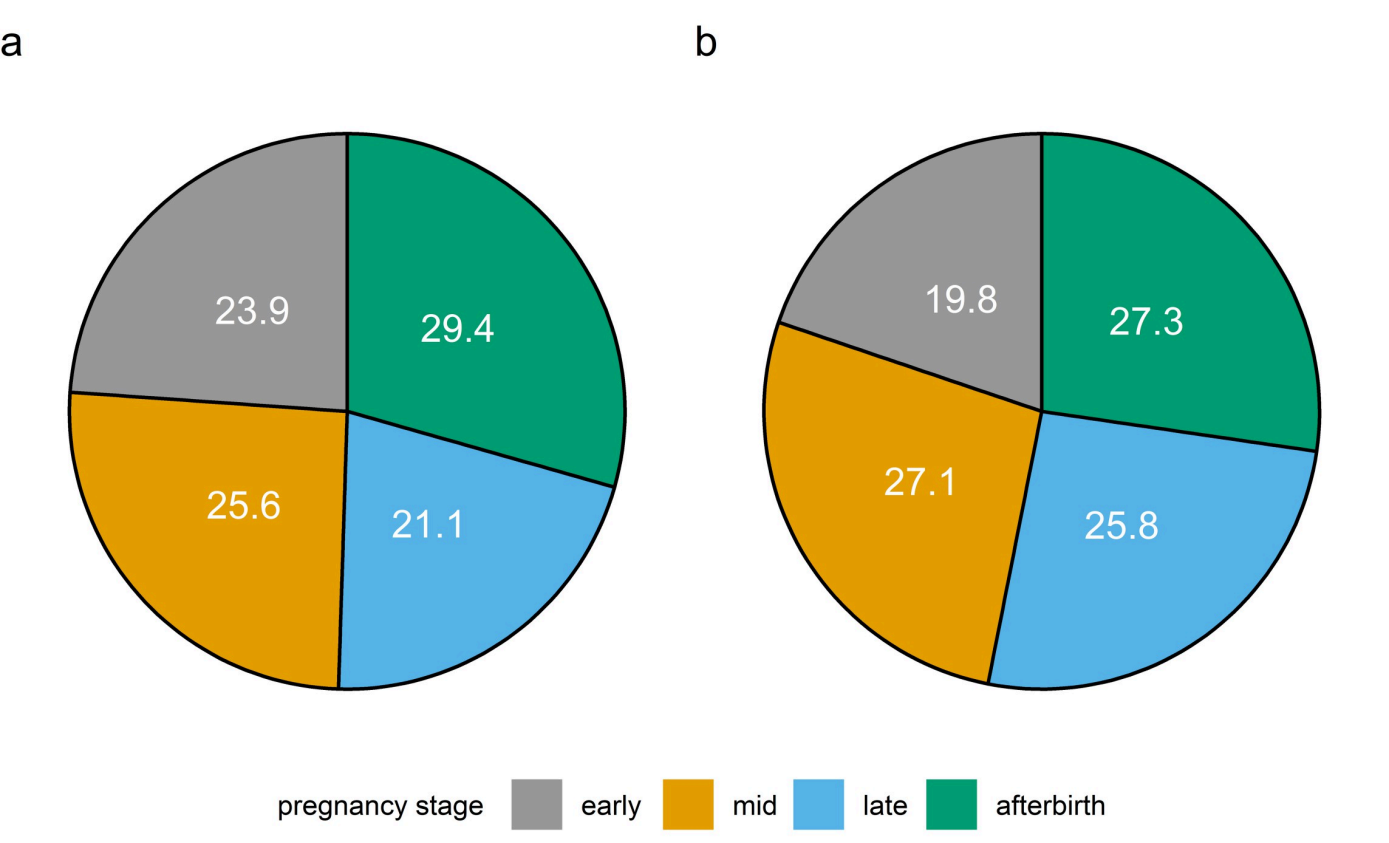
Relative proportions among the different pregnancy states for (a) glycosylated samples and (b) deglycosylated samples. Note the different basic population of Total Area Fragment (a = 4,774,159; b = 7,567,626).

Overall, the mean Total Area Fragment between glycosylated and deglycosylated samples increased by 58.5%. Distributions of the relative proportion of boPAG abundances at the different pregnancy stages for glycosylated samples and deglycosylated samples are shown in Fig 4. In glycosylated samples, we found an equal distribution of the relative proportions among the different pregnancy stages. Upon deglycosylation, the distribution is slightly shifted. The relative proportions of mean Total Area Fragment during mid and late pregnancy increased whereas the proportions during early pregnancy and post-partum decreased.

Furthermore, we observed changes in abundances and distribution patterns depending on the glycosylation status of the samples. The levels of boPAG 1 were significantly increased and the levels of boPAG 18 were significantly decreased in the deglycosylated samples during pregnancy and postpartum in comparison to the glycosylated samples (P < 0.001). The additionally observed differences in the abundance of other glycosylated and deglycosylated boPAGs did not reach statistical significance. Nevertheless, we discovered changes in the distribution pattern of the different boPAGs. The above mentioned boPAG 1 had increased relative abundances in deglycosylated samples during mid and late pregnancy compared to the glycosylated ones. BoPAG 2 exhibited a nearly equal distribution among glycosylated and deglycosylated samples with a slight increase in relative abundances in deglycosylated samples. BoPAG 3, boPAG 9 and boPAG 21 displayed similar distribution pattern between glycosylated and deglycosylated samples with a small decrease in relative abundances in deglycosylated samples. BoPAG 4, boPAG 6 and boPAG 8 showed a major change in their distribution pattern which was dependent on the glycosylation state of the samples. For those proteins, in the glycosylated samples highest abundances were observed in early and late pregnancy. Upon deglycosylation they exhibited decreasing levels throughout gestation. BoPAG 7 and boPAG 14 displayed distribution pattern in glycosylated samples which resembled the profile of boPAG 4, boPAG 6 and boPAG 8. However, in the deglycosylated samples they showed an increase in relative abundances during mid pregnancy. An opposite effect was monitored in regard to boPAG 17. Of note, boPAG 16 and boPAG 20 showed a decrease in relative abundance during mid pregnancy and a slight increase during late pregnancy when comparing glycosylated with deglycosylated samples. Overall, boPAG 18 had lower abundances in deglycosylated samples than in glycosylated ones. Another set of boPAGs comprised boPAG 5, boPAG 10, boPAG 11 and boPAG 15. These proteins displayed no glycosylation-dependent difference in their distribution.

**Fig 5:**
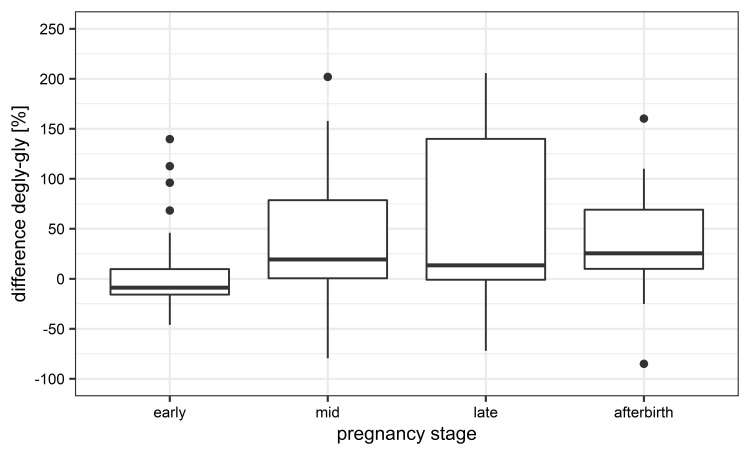
Box-and-whisker plot visualization of the percentage change of Total Area Fragment values from peptides between deglycosylated and glycosylated samples in the course of pregnancy and post-partum.

We observed major differences in the percentage change of Total Area Fragment values from peptides between deglycosylated and glycosylated samples during mid and late pregnancy as well as post-partum (Fig 5). One-way ANOVA indicated an effect of the pregnancy stage on the percentage change (P = 0.07). During early pregnancy the majority of the peptides showed changes in their Total Area Fragment in the range of 30%.

**Fig 6:**
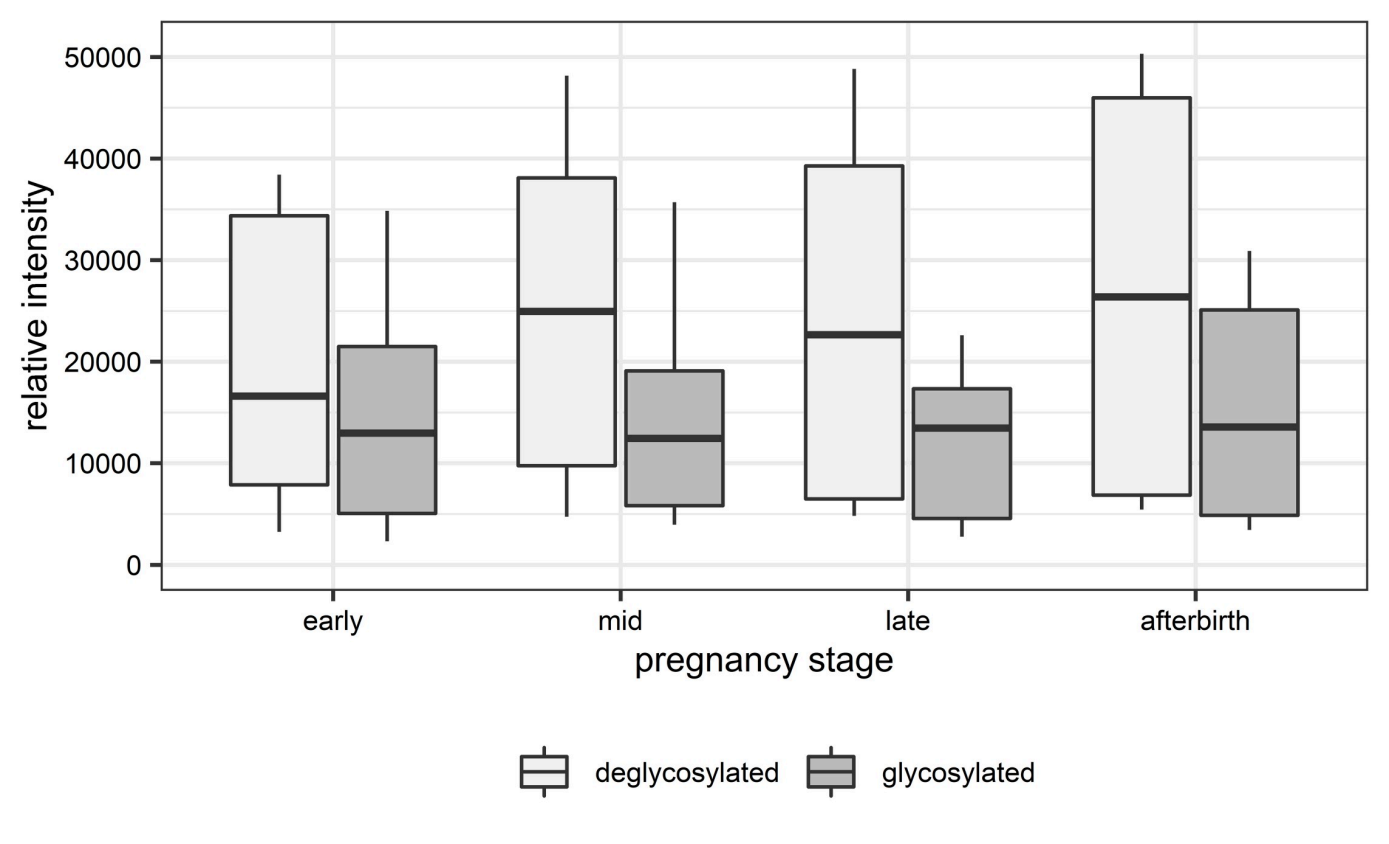
Overview of the results from Western blot analyses. Box-and-whisker plot visualization represents the intensities of the fluorescence signal.

These findings are further supported by the results of our Western blot analysis (S2 Fig in S2 File). Fig 6 summarizes the signal intensities of the six different boPAG-antisera within the different pregnancy stages and between the two sample types. The largest alterations in signal strength between deglycosylated and glycosylated samples could be observed during mid and late pregnancy as well as post-partum. Analysis with ANOVA showed a significant effect of glycosylation on signal intensities (P = 0.04). Further Bonferroni-corrected t-test revealed that binding of the six boPAG-antisera to deglycosylated samples is significantly (P = 0.03) enhanced over all investigated pregnancy stages. This suggests that binding from the polyclonal sera may be sterically hindered by the attached N-glycans, especially during mid pregnancy, late pregnancy and post-partum.

**Fig 7:**
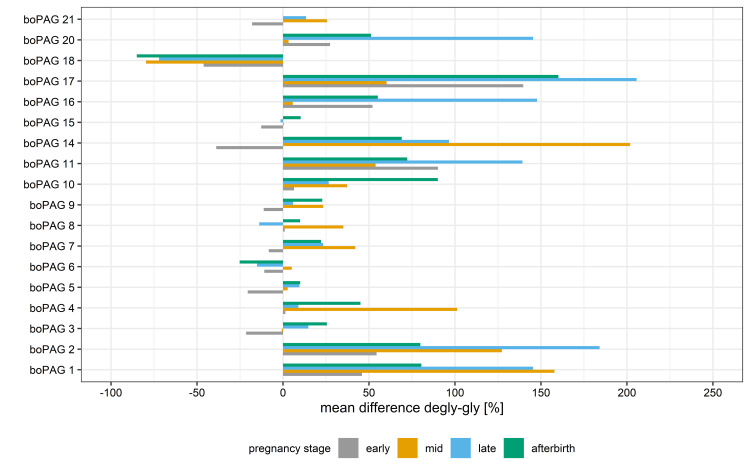
Mean percentage change of Total Area Fragment values from boPAGs between deglycosylated (degly) and glycosylated (gly) samples in the course of pregnancy and post-partum.

Based on the detected variations in the PRM assay, boPAGs were assigned to two different sets as a function of their percentage change within the different pregnancy stages (Fig 7). The first set included boPAG 3, boPAG 5, boPAG 6, boPAG 7, boPAG 8, boPAG 9, boPAG 15 and boPAG 21 which showed only minor differences with either an increase or a decrease in the two sample types in a range of 30% within a pregnancy stage. The second set comprised boPAG 1, boPAG 2, boPAG 4, boPAG 10, boPAG 11, boPAG 14, boPAG 16, boPAG 17 and boPAG 20. These boPAGs seemed to be strongly glycosylated during pregnancy with an increase in the Total Area Fragment between glycosylated and deglycosylated samples within a range of 35.1% - 205.6%. The only exception of this grouping is the peptide assigned to boPAG 18. Here we observed highly negative percentage changes within pregnancy and post-partum (early pregnancy -46.1%; mid pregnancy -79.6%; late pregnancy -72.1%; post-partum -85.0%).

## Discussion

The development of new mass spectrometers and data acquisition schemes for the characterization and quantification of proteins with high sensitivity have driven tremendous advances in proteomics over the past years [22–24, 43]. As a new targeted acquisition workflow, PRM has gained particular interest because of its precision and ease of method development [22, 24, 43, 48]. In this study, a PRM assay for the determination of the relative protein abundances of 18 different boPAGs during pregnancy and after calving was developed. Furthermore, we investigated glycosylated and deglycosylated samples to assess possible effects of the glycosylation status on the outcome of the applied analytical method and on changes in the levels of relative abundances.

There are only a few studies that give insights into the protein level of different boPAGs [12, 16–19, 21]. Zoli et al. (1991) [17] and Sousa et al. (2002) [21] identified boPAG 1 by N-terminal sequencing in a mix of cotyledon tissue derived from 2- to 6-month-gestations [17] and in cotyledon tissue isolated from zebus at 3 different gestational ages (10–11 weeks; 20–21 weeks; 30–31 weeks) [21]. In 2005 the first mass spectrometric analysis of boPAGs were conducted by two independent research groups. Green et al. (2005) identified boPAG 4, boPAG 6, boPAG 7, boPAG 16, boPAG 17, boPAG 20 and boPAG 21 by MALDI-TOF mass spectrometry with the peptide mass fingerprinting (**PMF**)-method from two placental extracts (obtained from cotyledons of 18 cm and 40 cm crown-rump fetuses) [18]. Klisch et al. (2005) purified different boPAGs (boPAG 1, boPAG 6, boPAG 7, boPAG 17) from cotyledonary tissue of different mid pregnancy placentas (day 100; day 155; day 180) and analyzed them by MALDI-TOF/TOF mass spectrometry with same method as Green et al. (2005) [16, 18]. No differences were detected between the different examined gestational stages. The most intensive band (66 kDa) on a Coomassie blue stained gel was identified as boPAG 1 [16]. Taken together, these results indicate that boPAG 1 is probably the most abundant boPAG in cotyledonary tissue, especially during mid-pregnancy. This is in accordance with our results as we also identified boPAG 1 as the most abundant boPAG during pregnancy. Additionally, our PRM assay also detected the other boPAGs found in the studies of Klisch et al. (2005) [16] and Green et al. (2005) [18] in the respective gestational stages.

The study of Touzard et al. (2013) investigated three different boPAG profiles (boPAG 1; boPAG 2; boPAG12) during gestation using Western blot analysis [12]. Therefore, they generated specific antibodies against the respective boPAGs which were then used to examine the expression levels of boPAGs in cotyledonary and intercotyledonary regions in 60 to 220-day-old bovine placentas [12]. Our protein profiles of the respective boPAGs in glycosylated samples showed high conformity in terms of the distribution pattern, when looking at results of the cotyledonary samples from day 80 onwards. Touzard et al. (2013) found highest abundance levels of boPAG 1 in early pregnancy (day 80) and a slight decrease of protein levels from early to mid (day 100) and late pregnancy (day 220). BoPAG 2 exhibited clearly a difference between protein levels from early pregnancy (day 80) in comparisons to protein levels in mid (day 100) and late pregnancy (day 220) and protein abundance of boPAG 11 remained at nearly same abundance levels during all gestation periods which were considered in this study [12]. Furthermore, Touzard et al. (2013) could not detect significant differences on protein level for the respective boPAGs in samples collected at day 80, day 100 and day 220 [12]. The only discrepancy between our study and the study conducted by Touzard et al. (2013) [12] are the lower protein levels of boPAG 1 and boPAG 2 observed in samples collected at day 60 compared to the ones obtained at the early pregnancy stage in our study. The reasons for the differences are probably the range of our early pregnancy stage compromising samples from 35–90 days of gestation in comparison to a specific sample timepoint and the use of a different antibody-based method in the study of Touzard et al. (2013) [12].

In summary, the comparison of our findings with the few existing proteomic studies on boPAGs shows that the developed PRM assay provides reliable and comparable results. Furthermore, we were able to expand the existing knowledge of previous protein analysis studies on boPAGs due to the advantages of PRM.

The available body of literature shows that boPAGs can have up to six potential N-glycosylation sites and that the degree of N-glycosylation seems to be the major factor in boPAG molecular mass [4, 12, 16]. N-glycans are usually attached to the amido group of an N side chain in a particular consensus sequence (NxS or NxT where x ≠ P) but such sequences may be glycosylated only partially or not at all [45, 49]. It is known that N-glycans are involved in important cellular processes including cell-cell and receptor ligand interactions, immune response or apoptosis [46, 50]. All those functions are also discussed for boPAGs during placentation and ongoing pregnancy highlighting the important functional role of the attached N-glycans in these proteins [1, 11, 19]. However, despite the obvious biological importance, our knowledge of in vivo N-glycosylation sites and the regulation of boPAG-glycosylation during pregnancy is still very limited [19]. For the stated reasons and the possibility to detect a signal shift between glycosylated samples and deglycosylated samples with modern LC-MS technologies [46, 47], we decided to examine the effect of glycosylation in our study.

Enzymatic digestion of all samples with PNGase F resulted in changes of relative boPAG abundances and of their distribution pattern. The overall mean of the Total Area Fragment between glycosylated and deglycosylated samples increased by 58.5%. This increase in Total Area Fragment was not equally distributed over the different boPAGs and pregnancy stages. In our study, the largest influence of glycosylation was detectable in mid and late pregnancy samples as well as post-partum. These results were further verified by immunoblotting. As already described, the detected band signals in all investigated samples shifted their apparent molecular weights after PNGase F treatment [12, 16, 19] and we found a much more intensive binding of the six different boPAG antisera to the deglycosylated samples compared to the glycosylated samples. However, this method is limited to the analysis of proteins with available antisera and can only identify protein-wide glycosylation occupancy [47]. Western blotting is not able to distinguish the extent of glycosylation of different boPAGs and at different pregnancy stages. PRM can overcome both of these limitations and provides an analysis tool that can be used for site-specific analysis of protein glycosylation [47, 48]. PRM-based protein assays do not require an antibody and have the advantage of multiplexed detection of analytes [48].

As a result, we were able to divide the boPAGs into groups. One group consisting of eight boPAGs (boPAG 3; boPAG 5; boPAG 6; boPAG 7; boPAG 8; boPAG 9; boPAG 15; boPAG 21) showed only minor differences with either an increase or a decrease between the two sample types within a pregnancy stage in a range of 30%. It seems likely that these differences arose from the normal variability of the measurement, especially for the boPAGs with a low abundance. The change in the distribution pattern of the relative protein abundances between glycosylated and deglycosylated samples in this set of boPAGs originate in the greater increase in the Total Area Fragment of the other boPAG group within the different pregnancy stages. It seems quite likely that the boPAGs of this group are not heavily glycosylated in any pregnancy stage.

The other group of boPAGs (boPAG 1; boPAG 2; boPAG 4; boPAG 10; boPAG 11; boPAG 14; boPAG 16; boPAG 17; boPAG 20) showed an increase in the Total Area Fragment between glycosylated and deglycosylated samples by 35.1%-205.6%. It seems that the second set of boPAGs have a higher or more complex degree of glycosylation during gestation compared to the first set and the deglycosylation process leads to a better detection of boPAGs in this group [45].

Given these data we asked if there is any correlation between group belonging and the number of potential glycosylation sites or the distance between potential glycosylation sites and the monitored proteotypic peptides. After sequence analysis (S1 File), we could not find such type of correlation. Furthermore, both groups consist of boPAGs which are monitored by proteotypic peptides with potential glycosylation sites. The lack of this relationship can have different reasons. It is known that the canonical glycosylation sequon is not an adequate predictor of glycosylation [47]. Only 70% of sequons carry a N-glycan and there is experimental evidence for N-glycosylation on consensus sequences different from the canonical one [46, 47, 50]. Additionally, the biochemical properties of the amino acid immediately proximal to the glycosylated N, the presence of either a NxS sequon or NxT sequon and the position of an asparagine within its protein sequence contributes to the extent or probability of glycosylation [47, 50–52]. Therefore, proof that a potential N-glycosylation site is occupied by a glycan requires experimental evidence [50]. A good example of the discrepancy between the existence of potential glycosylation sites and experimental proof is boPAG 2. The protein sequence of boPAG 2 consist of 6 sequons. Touzard et al. (2013) used a progressive enzymatic N-deglycosylation protocol with PNGase F to determine the number of occupied glycosylation sites of boPAG 1, boPAG 2 and boPAG 11 from late pregnancy placenta (day 220) by Western blot [12]. The digestion of the immunoreactive boPAG 2 protein indicated, that boPAG 2 has only one occupied N-glycosylation site in the respective pregnancy stage. Touzard et al. (2013) hypothesized that this result could have been related to folding of the protein [12]. The results from our PRM assay indicate that boPAG 2 seems to be glycosylated at least at one glycosylation site during early pregnancy and at two glycosylation sites during mid and late pregnancy and after gestation. In this study boPAG 2 was monitored by two proteotypic peptides. Both of them have a potential N-glycosylation site in their sequence. The peptide NYLDTAYVGNITIGTPPQEFR showed a decrease of the Total Area Fragment in early pregnancy sample (-27.2%) and an increase in mid (137.8%), late (139.3%) and afterbirth (110.0%) samples. The other peptide TFNPQNSSSFR showed an increase of the Total Area Fragment over all pregnancy stages (early pregnancy: 96.1%; mid pregnancy: 123.6%; late pregnancy: 199.2%; afterbirth: 76.1%). Altogether this supports the findings of different studies which show that boPAG glycosylation undergoes major changes during pregnancy [12, 19, 20]. Nevertheless, the role of these observed changes and the mechanism of glycosylation in boPAGs remain unclear [19, 20] and more research is needed.

The PRM assay developed and described in this study provides a practical and efficient method that promises to be a powerful tool for further research on boPAGs. With this assay it is possible to compare protein abundances between cotyledons and intercotyledonary chorion during gestation and at term. In combination with a stepwise deglycosylation of the samples this might improve our knowledge of the mechanisms behind the observed changes of boPAG glycosylation. Furthermore, the PRM assay can be adapted to blood and milk samples. Accurate quantification of boPAGs in these body fluids is achievable by any of the commonly used stable isotope labelling techniques [26, 43, 48, 53]. This information provides insights into which boPAGs are released in the maternal circulation. This knowledge can be utilized for the development of new pregnancy detection systems. Güzel et al. (2018) showed that PRM can be used as an attractive alternative for immunoassay [53]. Therefore, the PRM assay itself can be applied as sensitive and reliable tool for pregnancy detection based on quantitation of 18 different boPAGs in blood or milk.

## Conclusions

In conclusion, we developed a PRM assay for the determination of the relative protein abundances of 18 different boPAGs during pregnancy and after calving. To our knowledge, this is the first study which addresses the detection of the different boPAGs in parallel in the time of pregnancy and afterbirth samples on protein level, thereby investigating the influence of glycosylation.

The detected boPAG distribution pattern in glycosylated samples confirmed the results of other proteomic studies. Highest degrees of glycosylation appeared in mid and late pregnancy samples as well as in afterbirth samples. Additionally, we identified a group of boPAGs that seems not heavily glycosylated in any pregnancy stage. A linkage between the impact of glycosylation and potential N-glycosylation sites or phylogenetic relation was not detected. The PRM assay itself and the results of this study give new starting points to address further research on boPAGs to better understand the physiological role during pregnancy and achieve a real knowledge of these proteins and their posttranslational modifications. For these reasons, the designed assay shall be improved and applied for the detection of individual boPAGs in maternal blood and milk in the near future. This improvement will also allow us to efficiently screen higher numbers of animals. Reliable identification and quantification will be ensured by the use of labelled synthetic peptides.

## Supporting information

S1 Raw images (PDF)

S1 File (PDF)

S2 File (PDF)

S3 File (PDF)
