# Supplementary material for: A multiplexed parallel reaction monitoring assay to monitor bovine pregnancy-associated glycoproteins throughout pregnancy and after gestation
Source: PLoS One. 2022 Sep 23;17(9):e0271057. doi: 10.1371/journal.pone.0271057 (PMC9506649; doi:10.1371/journal.pone.0271057)
Supplement: S1 Raw images — (PDF) [file pone.0271057.s001.pdf]

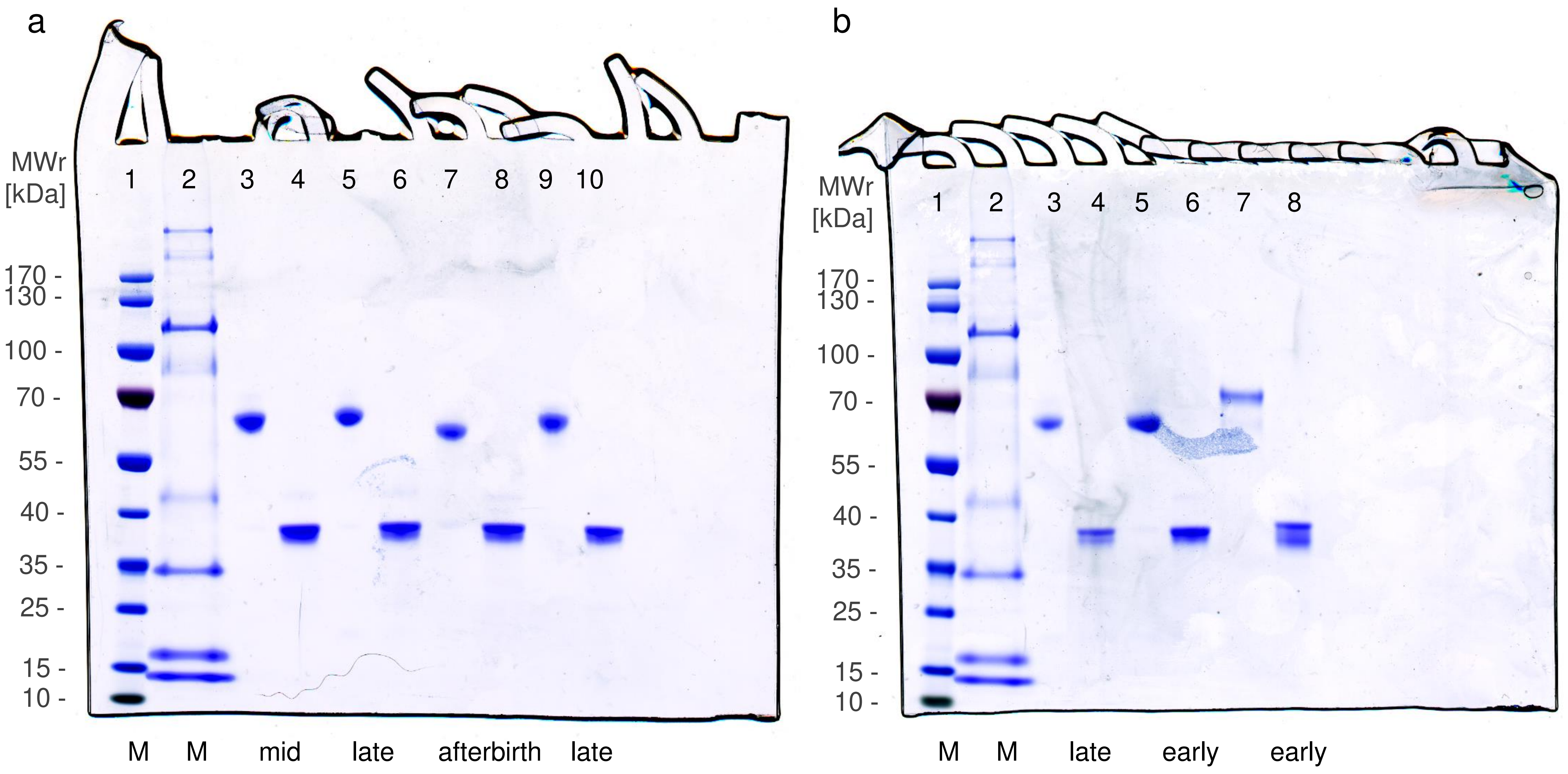

a
MWr [kDa]
1 2 3 4 5 6 7 8 9 10
170 -
130 -
100 -
70 -
55 -
40 -
35 -
25 -
15 -
10 -
M M mid late afterbirth late
b
MWr [kDa]
1 2 3 4 5 6 7 8
170 -
130 -
100 -
70 -
55 -
40 -
35 -
25 -
15 -
10 -
M M late early early

IgG 1421

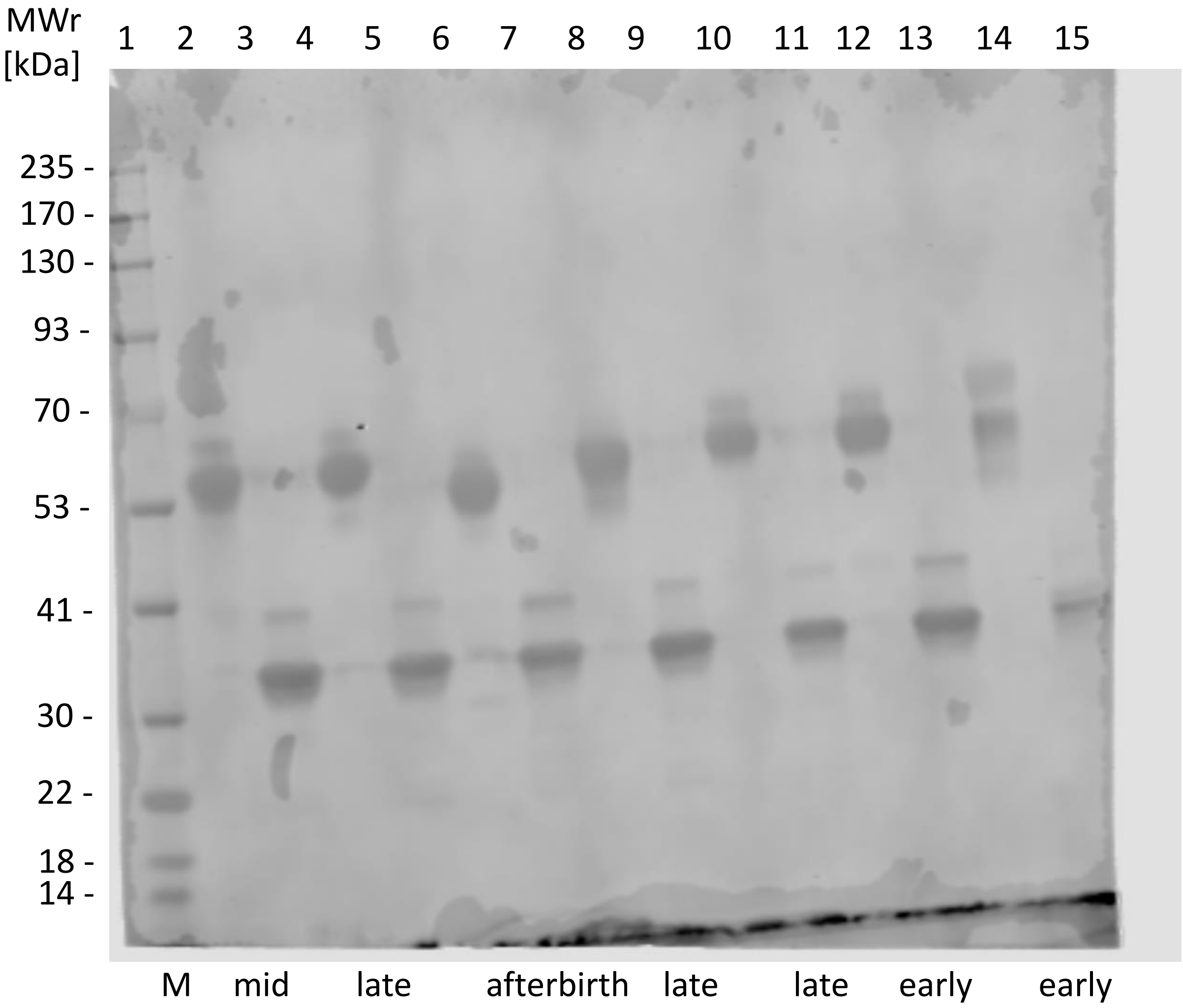
MWr
[kDa]
1 2 3 4 5 6 7 8 9 10 11 12 13 14 15
235 -
170 -
130 -
93 -
70 -
53 -
41 -
30 -
22 -
18 -
14 -
M mid late afterbirth late late early early

IgG 1428

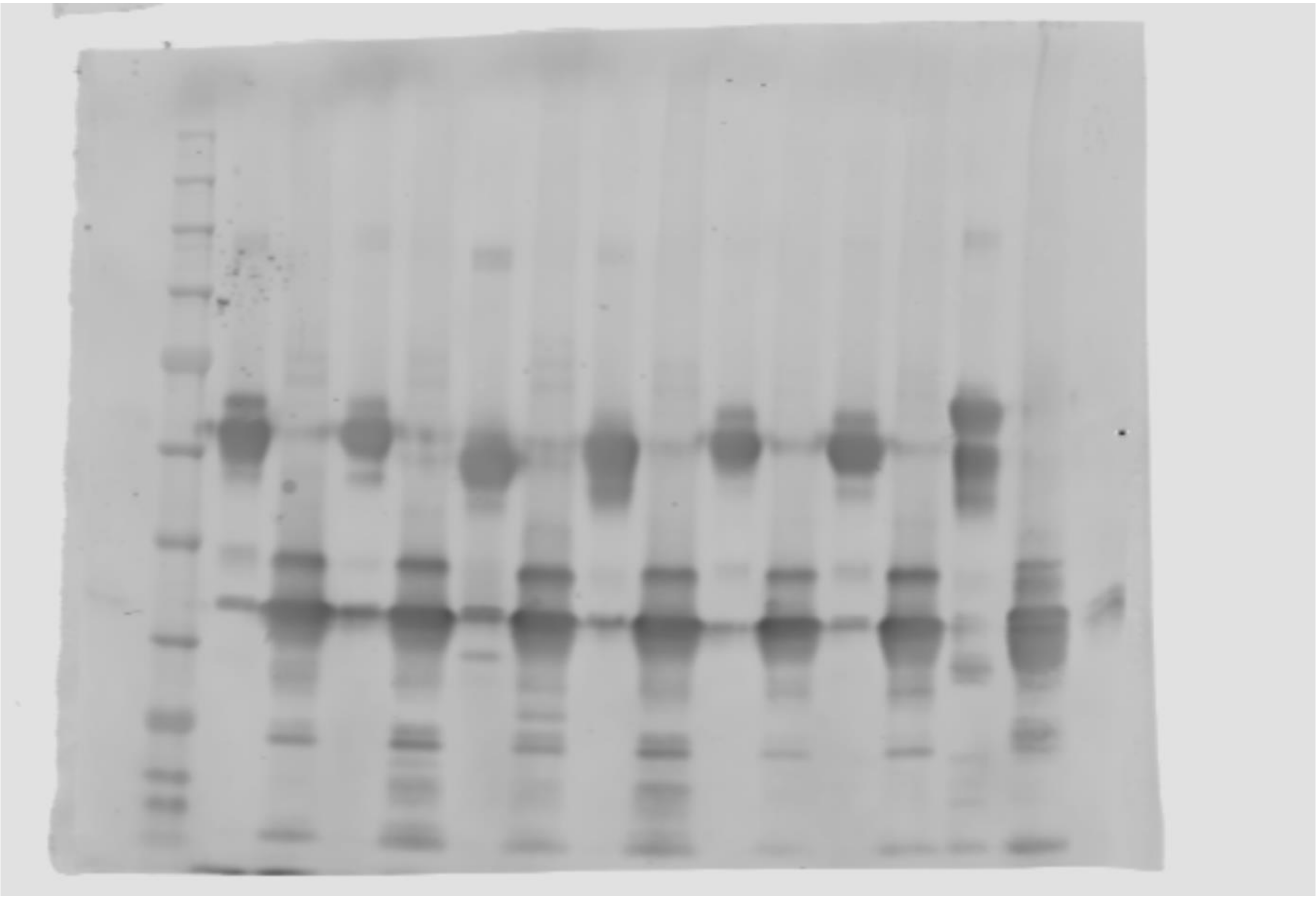
MWr
[kDa]
1 2 3 4 5 6 7 8 9 10 11 12 13 14 15
235 -
170 -
130 -
93 -
70 -
53 -
41 -
30 -
22 -
18 -
14 -
M mid late afterbirth late late early early

IgG 1429

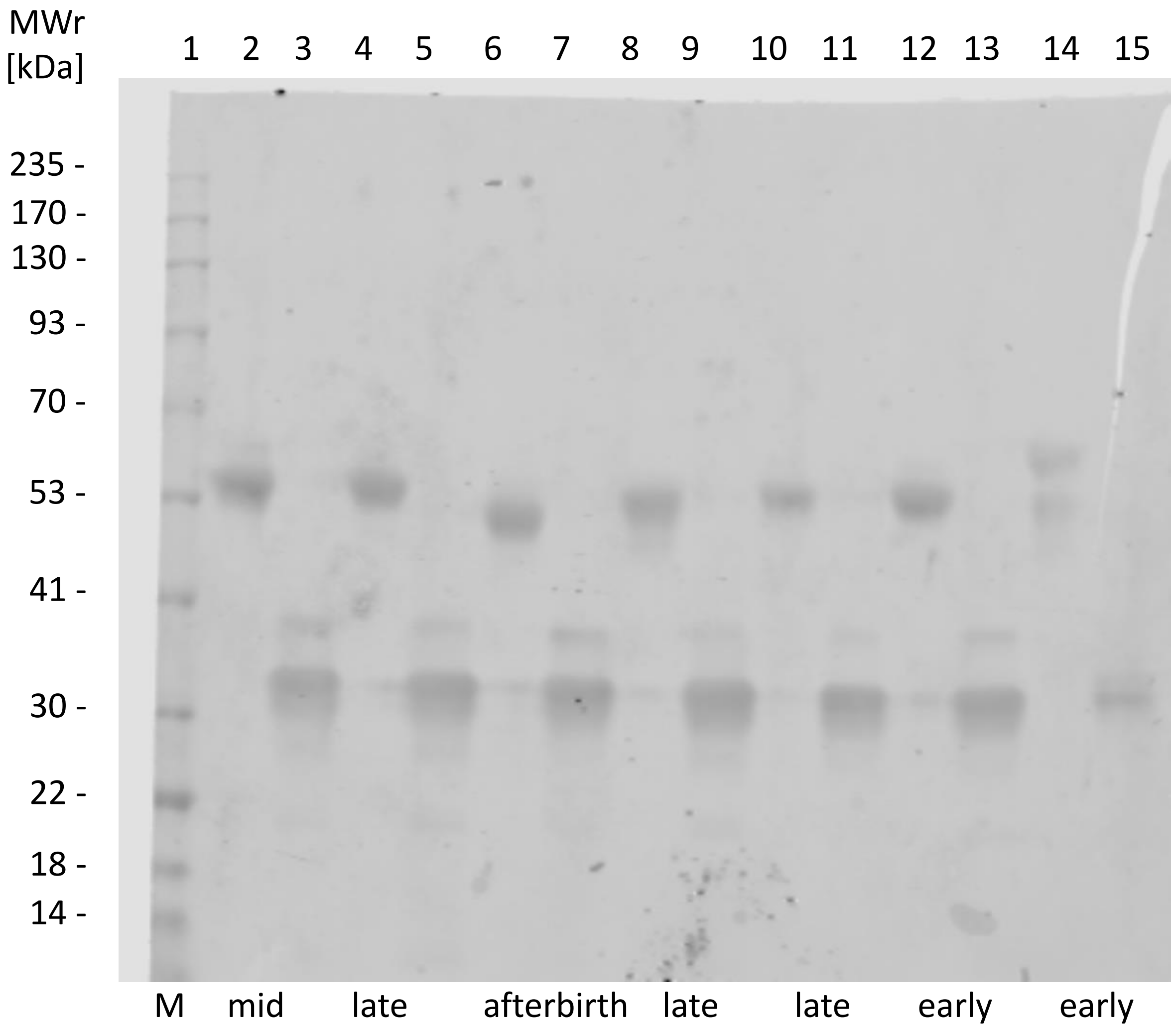
MWr
[kDa]
1 2 3 4 5 6 7 8 9 10 11 12 13 14 15
235 -
170 -
130 -
93 -
70 -
53 -
41 -
30 -
22 -
18 -
14 -
M mid late afterbirth late late early early

IgG 1432

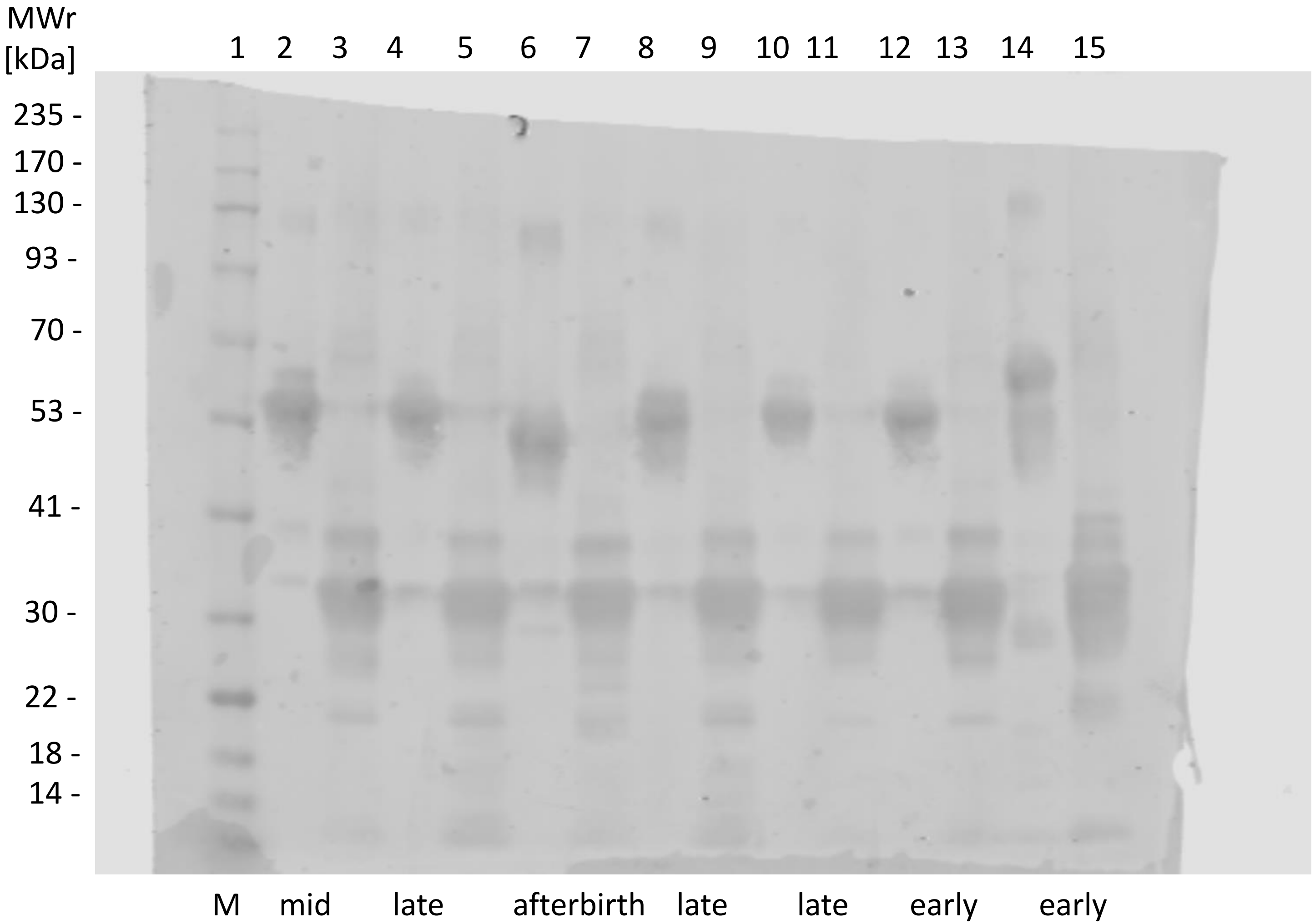
MWr
[kDa]
1 2 3 4 5 6 7 8 9 10 11 12 13 14 15
235 -
170 -
130 -
93 -
70 -
53 -
41 -
30 -
22 -
18 -
14 -
M mid late afterbirth late late early early

IgG 1438

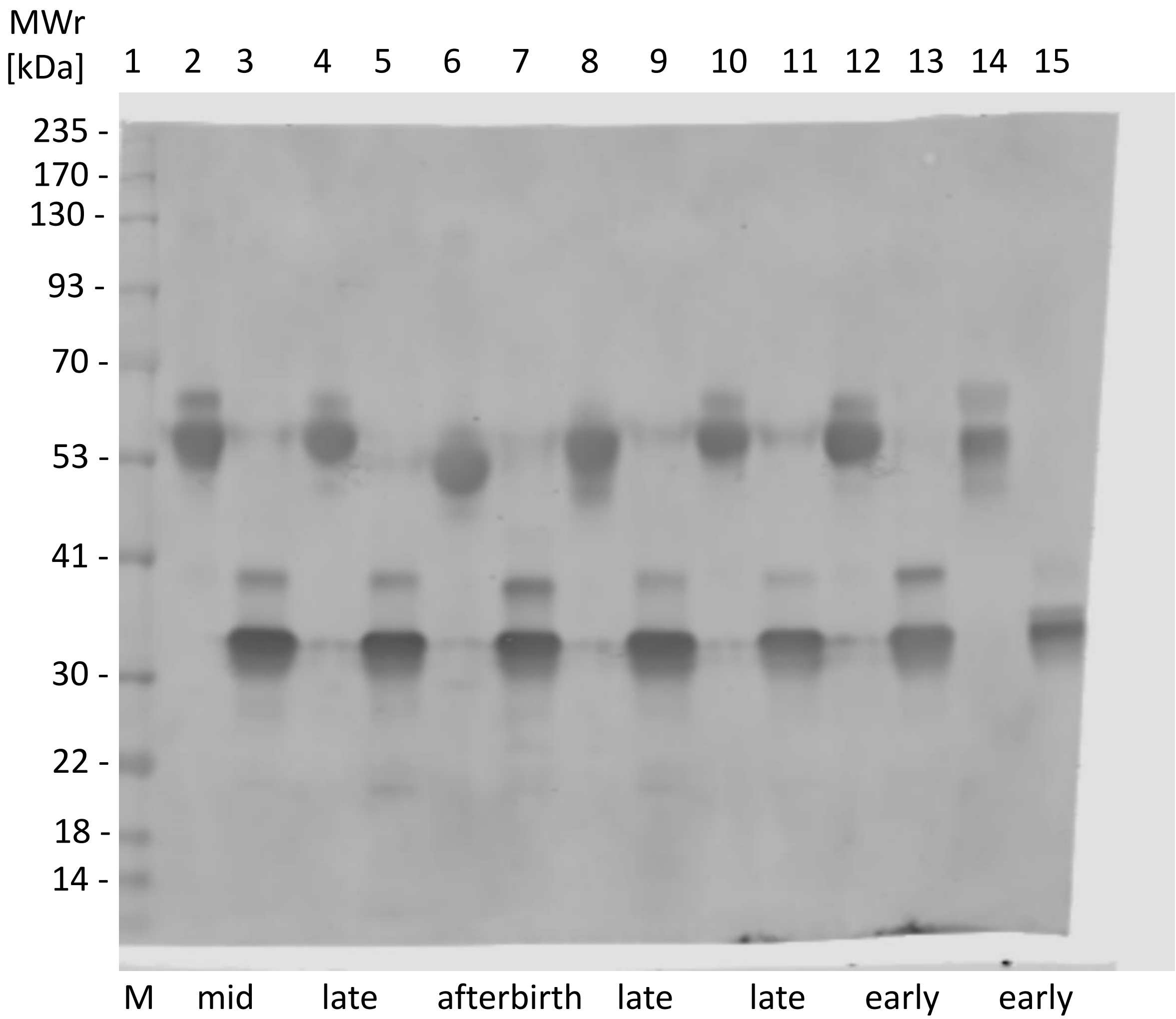
MWr
[kDa]
1 2 3 4 5 6 7 8 9 10 11 12 13 14 15
235 -
170 -
130 -
93 -
70 -
53 -
41 -
30 -
22 -
18 -
14 -
M mid late afterbirth late late early early

IgG 1440

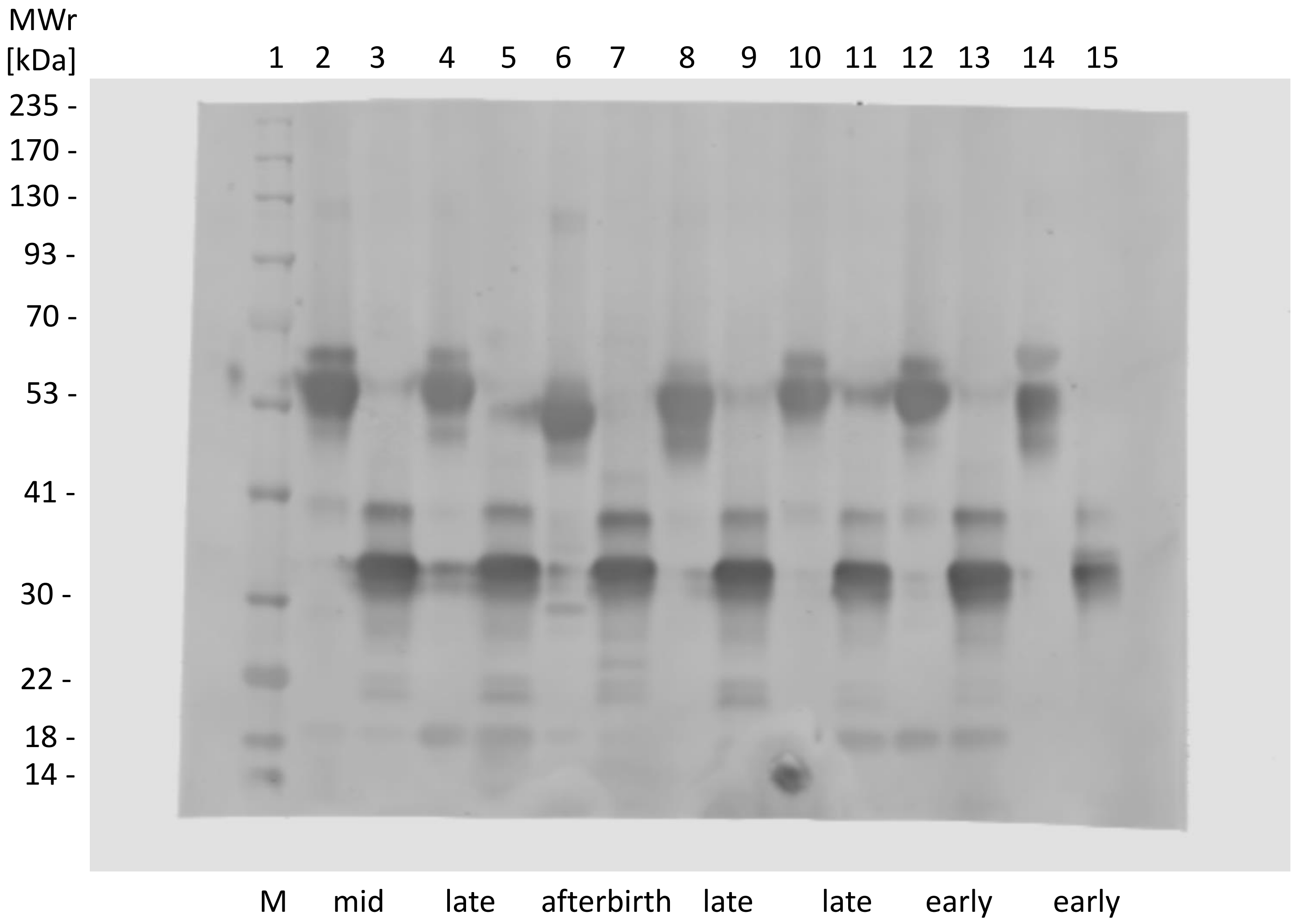
MWr
[kDa]
1 2 3 4 5 6 7 8 9 10 11 12 13 14 15
235 -
170 -
130 -
93 -
70 -
53 -
41 -
30 -
22 -
18 -
14 -
M mid late afterbirth late late early early
