# Supplementary material for: A multiplexed parallel reaction monitoring assay to monitor bovine pregnancy-associated glycoproteins throughout pregnancy and after gestation
Source: PLoS One. 2022 Sep 23;17(9):e0271057. doi: 10.1371/journal.pone.0271057 (PMC9506649; doi:10.1371/journal.pone.0271057)
Supplement: S1 File — (PDF) [file pone.0271057.s002.pdf]

BoPAG sequence data S1. Sequence Analysis of the 18 different boPAG

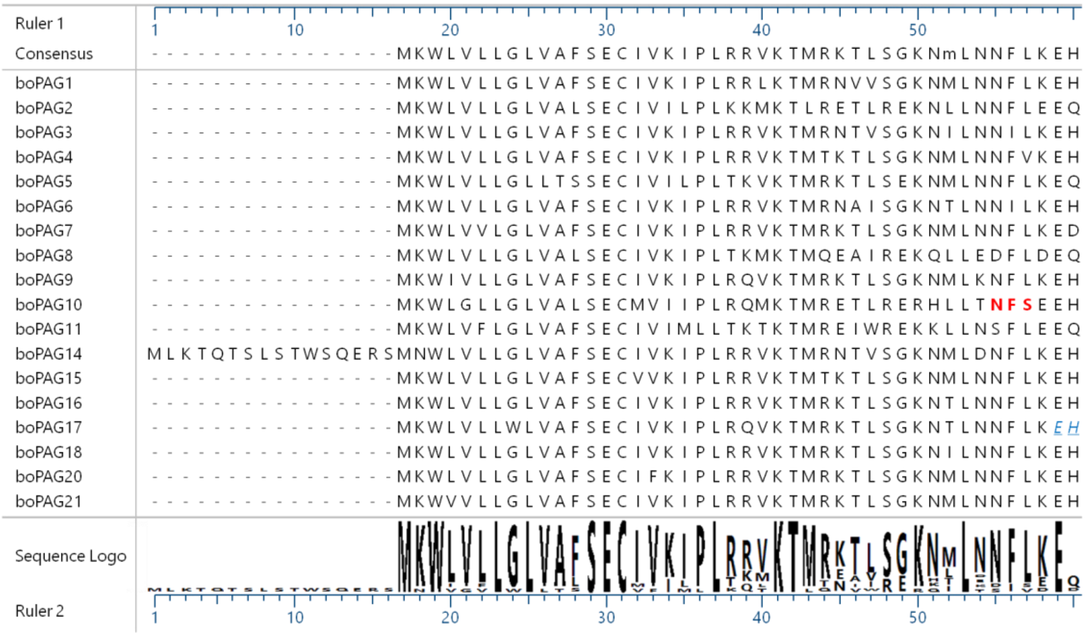

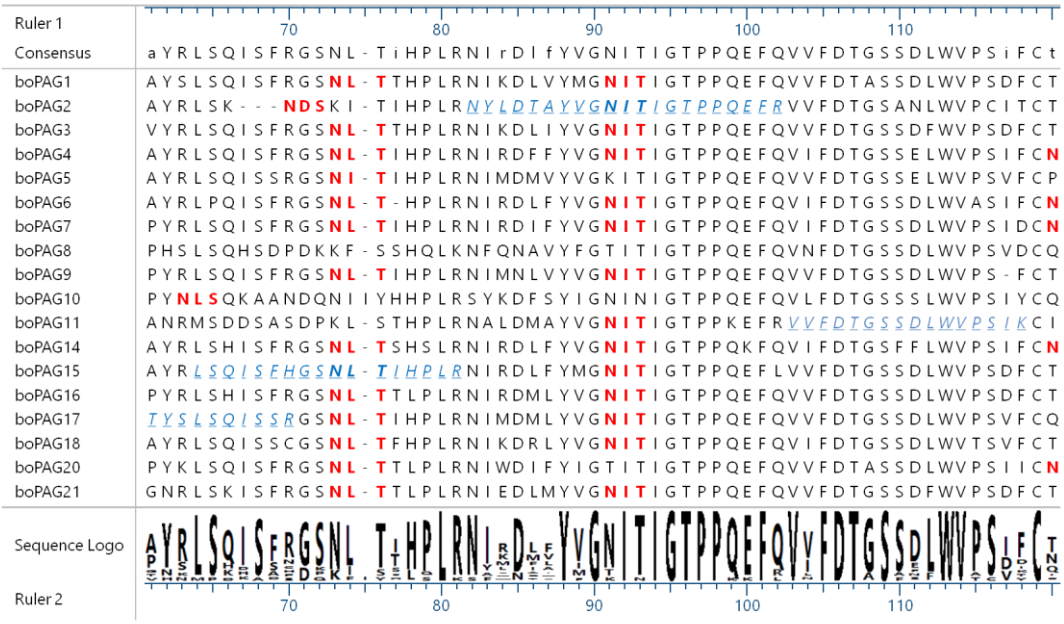

Red Markings: N-glycosylation sequons

Blue Markings: proteotypic peptides (listed in Table 1)

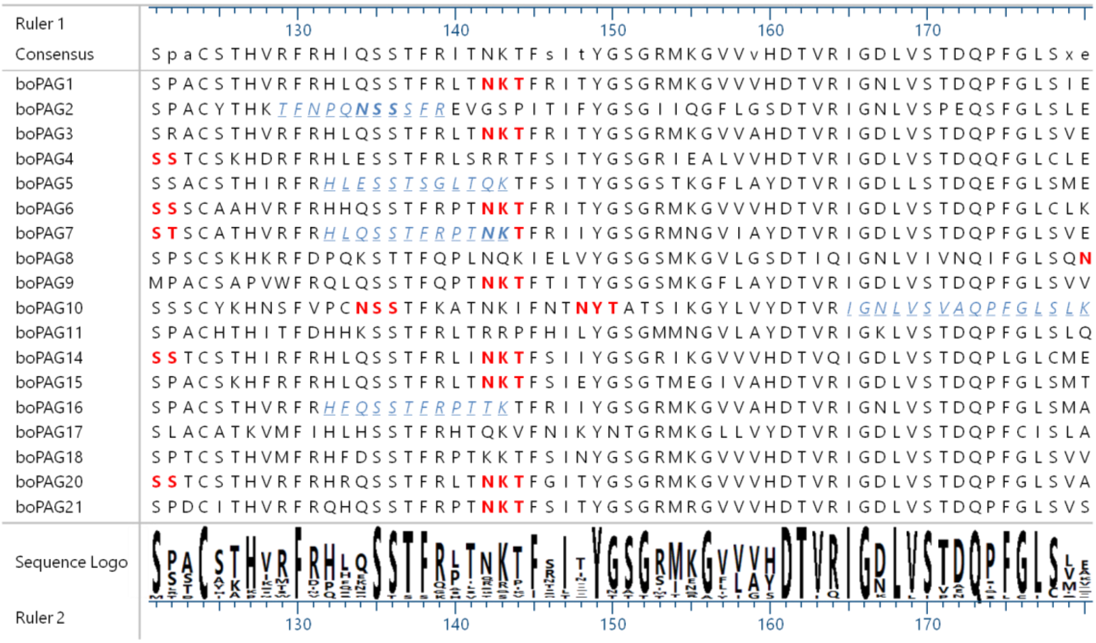

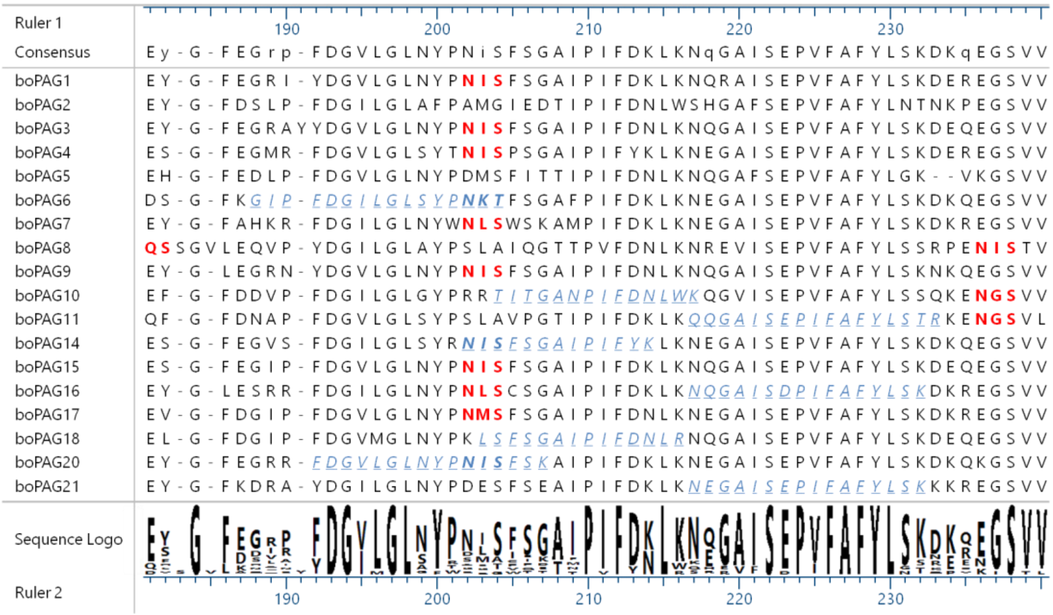

Red Markings: N-glycosylation sequons

Blue Markings: proteotypic peptides (listed in Table 1)

	250 / 260 / 270 / 280 / 290 (Ruler 1)
Consensus	MFGGVDHRYYKGELNWVPLIQAGDWsVHMDRISMKRKVIACSdGCeALVDTGTSIIvGPr
boPAG1	MFGGVDHRYYEGELNWVPLIQAGDWSVHMDRISIERKIIACSDGCKALVDTGTSDIVGPR
boPAG2	MFGGVDHRYYKGELNWIPVSQTSHWQISMN**NIS**M**NGT**VTACSCGCEALLDTGTSMIYGPT
boPAG3	MFGGVDHRYYEGELNWVPLIEAGDWIVHMDRISMKRKIIACSGSCEAIVDTGTSAIEGPR
boPAG4	MFGGADHRYYKGELNWIPLMKAGDWSVHMDRISMKRKVIACSGGCK*ALVDTGSSDIVGPS*
boPAG5	MFGGVDHTYYKGELNWVPLIQAGEWSLHMDRISMKRKVIACSGGCEAFYDTGTSLILGPR
boPAG6	MFGGVDHRYYKGELNWVPLIQVGDWFVHMDRTTMKRKVIACSDGCKALVDTGTSDIVGPS
boPAG7	MFGGVDHRYYKGELK*WVPLIQAVDWSVHVDR*ITMNREVIACSEGCAALVDTGSSNIQGPR
boPAG8	MFGGVDHTYHKGKLQWIPVTQARFWQVAMSSMTMNGNVVGCSQGCQAVVDTGTSLLVGPT
boPAG9	MFGGVDHQYYK*GELNWIPLIEAGEWR*VHMDRISMKRTVIACSDGCEALVHTGTSHIEGPG
boPAG10	MFGGVNRAYYKGELNWVPVSQVGSWHINIDSISM**NGT**VVACKRGCQALLDTGTAFLRGPR
boPAG11	MLGGVDHSYHKGKLNWIPVSQTKSWLITVDRISMNGRVIGCEHGCEALVDTGTSLIHGPA
boPAG14	MFGGVDHRYYKGELKWVPLIRAGDWSVHMDRISMKRKIIACSDCCKALVDTGTSDIAGPR
boPAG15	MFGGVDHRYYKGELKWVPLIEAGDWIVHMDCISMRRKVIACSGGCEAVVDTGVSMIKGPK
boPAG16	MFGGVDHRYYKGELNWVPLIRAGDWIVHVDRITMKREVIACSDGCAALVDTGTSLIQGPG
boPAG17	MFGGVDHRYYKGELNWVPLIQAGGWTVHVDRISMKRKIIACSGGCEALVDTGTALIKGPR
boPAG18	MFGGVDHRYYKGELNWIPLIQAGDWSVHMDSISMKRKVIACSGGCKAVVDTGTSLIEGPR
boPAG20	MFGGVDHRYYKGELNWVPLIRAGDWSVHVDRITMKGEVIGCSDGCTAMVDTGSSNIQGPG
boPAG21	MFGGVDHRYYKGELNWVPLIEEGDWSVRMDGISMKTKVVACSDGCEAVVDTGTSLIKGPR

Sequence Logo

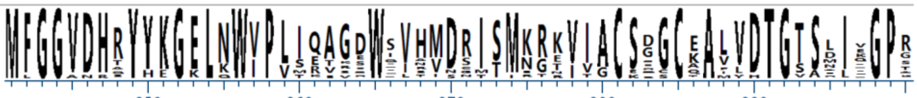

Ruler 2: 250 260 270 280 290

	310 / 320 / 330 / 340 / 350 (Ruler 1)
Consensus	RLVNNIqKLIGAtPR-GSeHYVSCSAVNTLPSIIFTINGINYPVPARAYILKDSRGrCYT
boPAG1	RLVNNIHRLIGAIPR-GSEHYVPCSEVNTLPSIVFTINGINYPVPGRAYILKDDRGRCYT
boPAG2	KLVTNIHKLMNARLE-NSEYVVSCDAVKTLPPVIFNINGIDYPLRPQAYIIKIQ-NSCRS
boPAG3	KLVNKIHKLIGARPR-HSKYYISCSAVNTLPSIIFTINGINYPVPGRAYVLKDSRGRCYS
boPAG4	*TLVNNIWK*LIGATPQ-GSEHYVSCSAVNSLPSIIFTIKSNNYRVPGQAYILKDSRGRCFT
boPAG5	RLVNNIQKLIGATPQ-GSEHYISCFAVISLPSIIFTINGINIPVPARAYIHKDSRGHCYP
boPAG6	TLVNNIWKLIRARPL-GPQYFVSCSAVNTLPSIIFTINGINYRLPARAYIHKDSRGRCYT
boPAG7	RLIDNIQRIIGATPR-GSKYYVSCSAVNILPSIIFTINGVNYPVPPRAYILKDSRGHCYT
boPAG8	HLVTDILKLINPNPILNDEQMLSCDAINSLPTLLLTINGIVYPVPPDYYIQRFSERICFI
boPAG9	RLVNNIHRLIRTRPF-DSKHYVSCFATKYLPSITFIINGIKYPMTARAYIFKDSRGRCYS
boPAG10	GSVSKIQKLIHARPI-DREHVVSCQAIGTLPPAVFTINGIDYPVPAQAYIQSLS-GYCFS
boPAG11	RPVTNIQKFIHAMPY-GSEYMVLCPVISILPPVIFTINGIDYSVPREAYIQKISNSLCLS
boPAG14	RLVNNIQKLIGSTPR-GSEYYISCSAVNTLPSIIFTINGINYPVPARAYILKDSRGRCYT
boPAG15	TLVDNIQKLIGATLR-GFKHYVSCSAVDTLPSITFTINGINYRVPARAYILKDSRGCCYS
boPAG16	RVIDNIHKLIGATPR-GSKHYVSCSVVNTLPSIIFTINGINYPVPAPAYILKDSRGYCYT
boPAG17	RLVNNIQKLIGTTPR-GSKHYVSCSVVNTLPSIIFTINGINYPVPARAYILKDSESNCYT
boPAG18	RLVNNIQKLIRAMPR-GSEYYVSCSAVNTLPPIIFTIKGINYPVPAQAYILKDSRGHCYT
boPAG20	RVIDNIHKLIGATPR-GSKHYVSCSAVSALPSVVFTINGINYPVPARAYVLKDFTGNCYT
boPAG21	KLVNKIQKLIGATPR-GSKHYVYCSAVNALPSIIFTINGINYPVPARAYILKDSRGRCYT

Sequence Logo

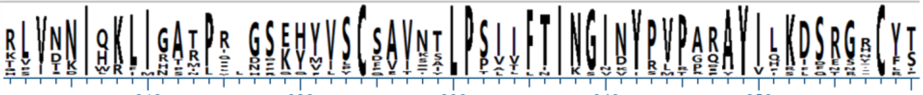

Ruler 2: 310 320 330 340 350

Red Markings: N-glycosylation sequons

Blue Markings: proteotypic peptides (listed in Table 1)

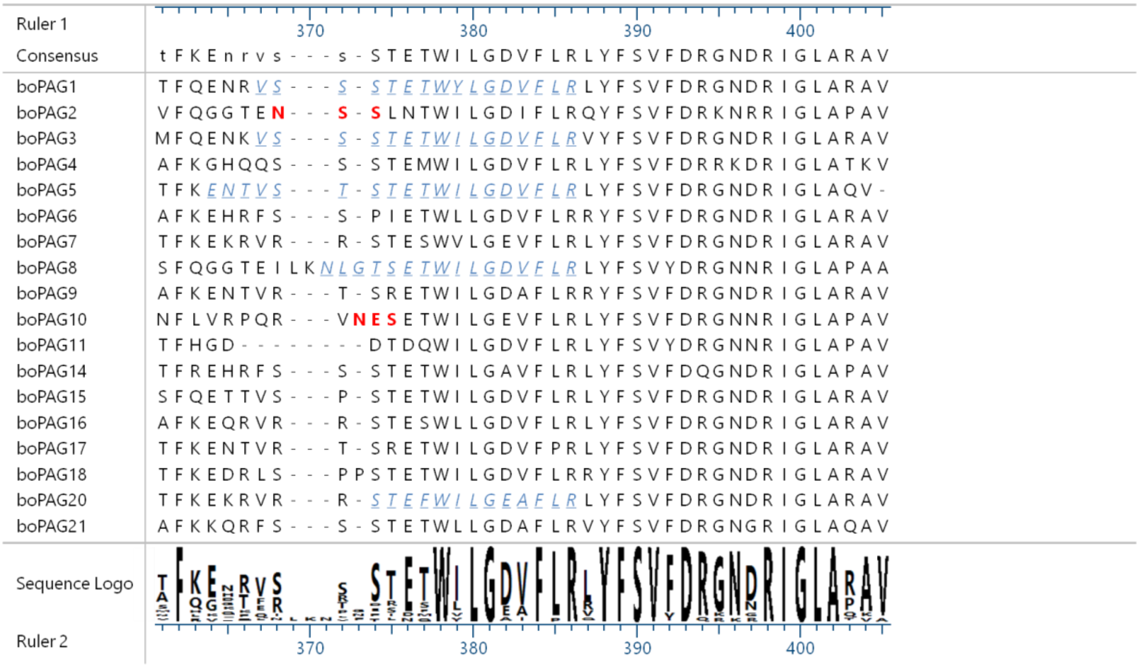

Red Markings: N-glycosylation sequons

Blue Markings: proteotypic peptides (listed in Table 1)
