# Supplementary material for: A multiplexed parallel reaction monitoring assay to monitor bovine pregnancy-associated glycoproteins throughout pregnancy and after gestation
Source: PLoS One. 2022 Sep 23;17(9):e0271057. doi: 10.1371/journal.pone.0271057 (PMC9506649; doi:10.1371/journal.pone.0271057)
Supplement: S2 File — (PDF) [file pone.0271057.s003.pdf]

SUPPLEMENTAL MATERIAL

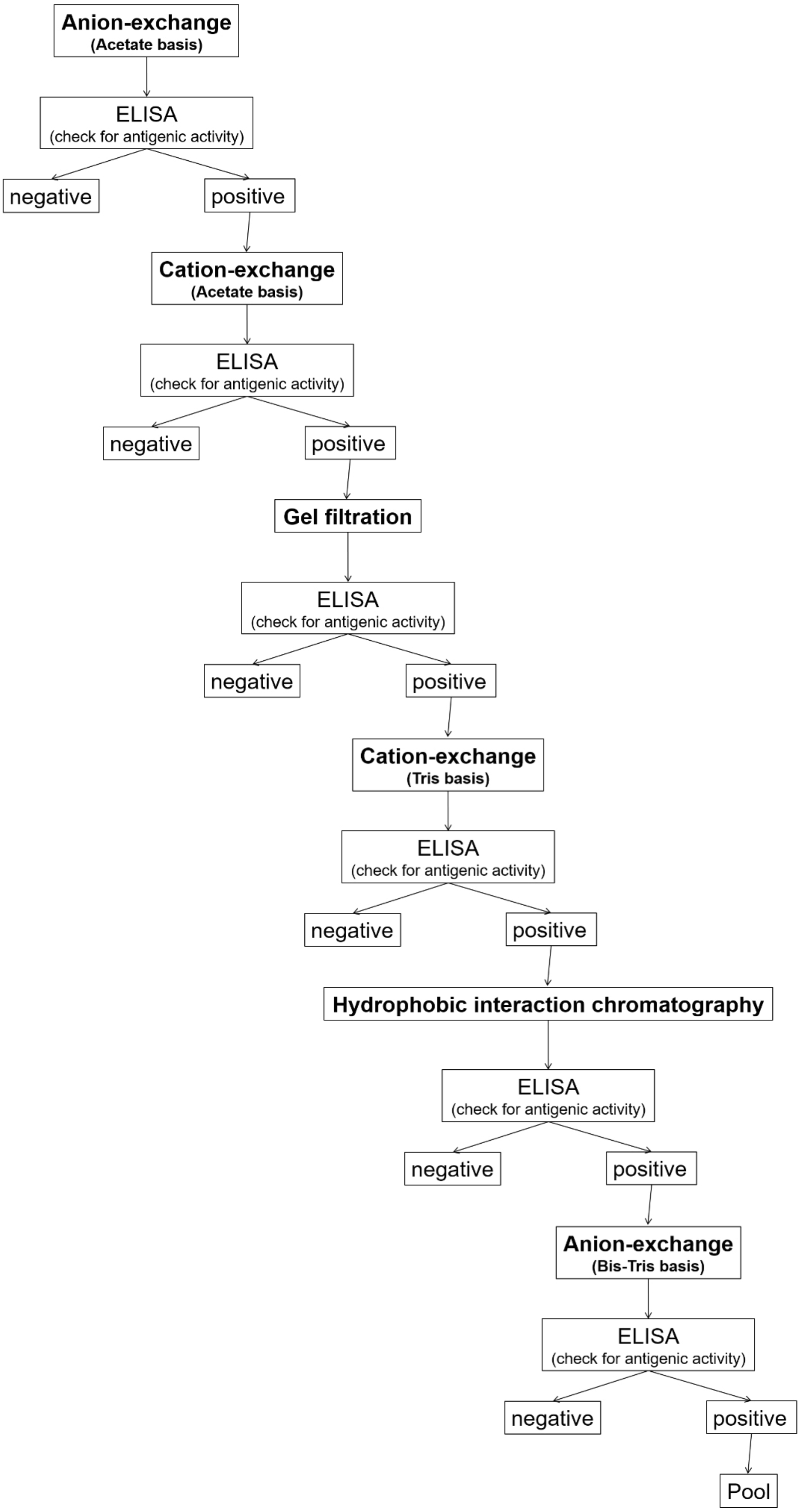

Fig S1. Overview of the FPLC-Workflow.

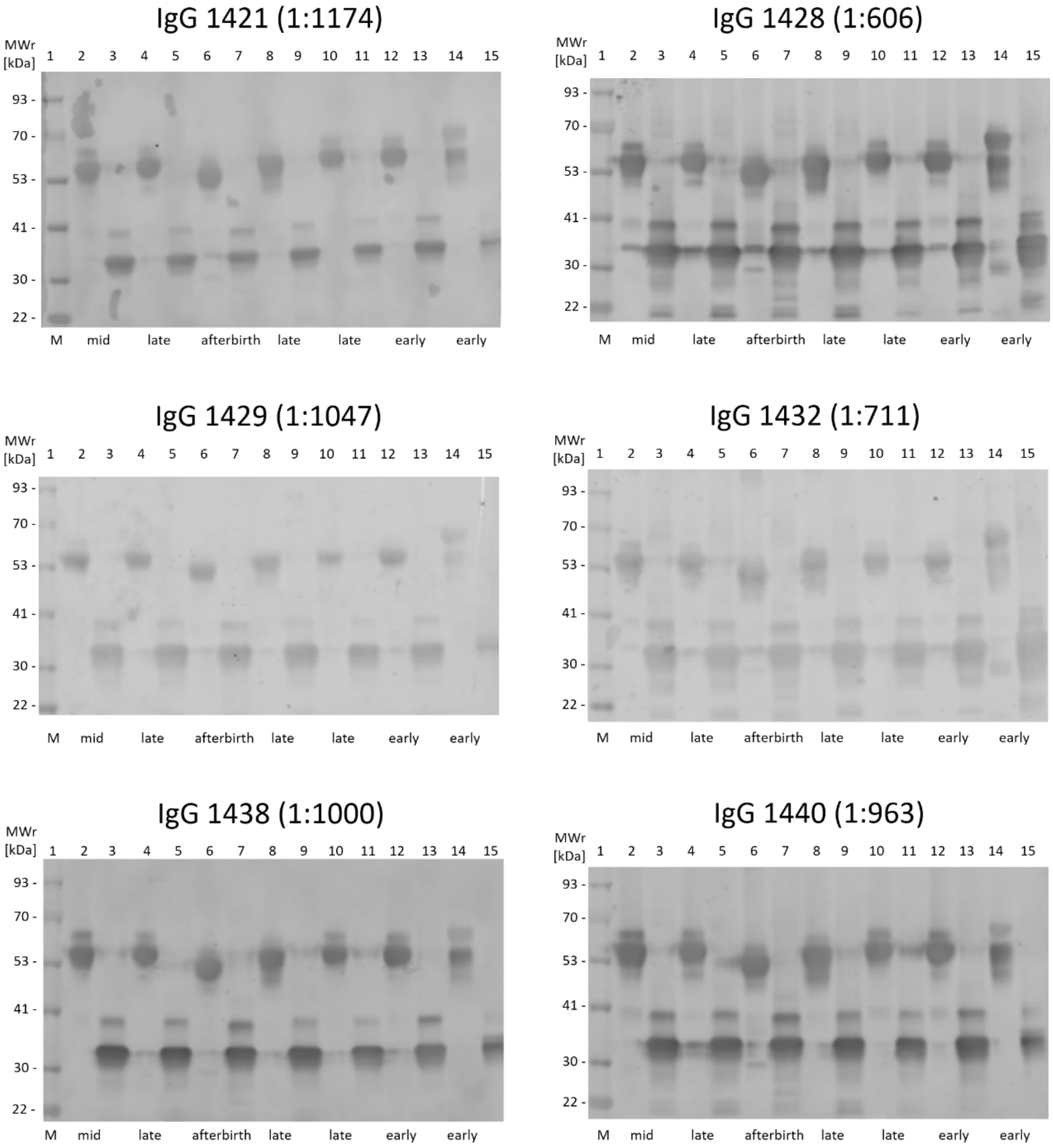

Fig S2. Overview of the six Western blots. Blots were probed with six different bovine Pregnancy-Associated Glycoprotein (boPAG) antisera (IgG 1421-IgG 1440). Dilutions are indicated inside the parentheses.

Lane 1: Marker
Lanes 2, 4, 6, 8, 10, 12, 14: glycosylated samples
Lanes 3, 5, 7, 9, 11, 13, 15: deglycosylated samples

The three late pregnancies and the two early pregnancies are represented by different samples as described in the results part. Samples of Lane 14 and Lane 15 were not analyzed by Mass Spectrometry. Relative intensity-values were analyzed for the 53 kDa band and above for glycosylated samples and between 30 kDa and 41 kDa for deglycosylated ones.
