# Supplementary material for: A multiplexed parallel reaction monitoring assay to monitor bovine pregnancy-associated glycoproteins throughout pregnancy and after gestation
Source: PLoS One. 2022 Sep 23;17(9):e0271057. doi: 10.1371/journal.pone.0271057 (PMC9506649; doi:10.1371/journal.pone.0271057)
Supplement: S3 File — (PDF) [file pone.0271057.s004.pdf]

List of identified boPAGs in the sample from mid gestation (for an SDS-PAGE gel image of the samples please see Figure 1a: Lane 3 and 4).

Table S1. List of identified boPAGs in the sample from mid gestation (glycosylated).

PAG	Protein Identification Probability [%]	Protein Percentage of Total Spectra [%]	Exclusive Unique Peptide Count	Exclusive Unique Spectra Count	Total Spectrum Count	Percentage of Amino Acids Identified [%]
PAG 1 (NP_776836.1)	100	0.80	19	38	2850	61.32
PAG 3 (NP_001291497.1)	100	0.11	1	3	401	48.03
PAG 4 (NP_788788.1)	100	0.11	7	8	384	28.42
PAG 6 (NP_788790.1)	100	0.11	7	9	407	41.42
PAG 7 (NP_001103448.1)	100	0.22	1	1	795	35.79
PAG 10 (NP_788794.2)	100	0.10	2	3	357	48.03
PAG16 (NP_788798.1)	100	0.22	5	5	781	42.42
PAG 20 (NP_788802.1)	100	0.21	1	1	731	49.47
PAG 21 (NP_788803.1)	100	0.16	1	2	579	10.26

Table S2. List of identified boPAGs in the sample from mid gestation (deglycosylated).

PAG	Protein Identification Probability [%]	Protein Percentage of Total Spectra [%]	Exclusive Unique Peptide Count	Exclusive Unique Spectra Count	Total Spectrum Count	Percentage of Amino Acids Identified [%]
PAG 1 (NP_776836.1)	100	0.87	21	37	3115	78.68
PAG 3 (NP_001291497.1)	100	0.15	1	2	528	41.99
PAG 4 (NP_788788.1)	99	0.13	0	0	462	27.89
PAG 6 (NP_788790.1)	100	0.15	5	6	543	30.87
PAG 7 (NP_001103448.1)	100	0.26	1	1	944	35.26
PAG 10 (NP_788794.2)	100	0.11	1	2	398	33.60
PAG16 (NP_788798.1)	100	0.25	3	3	890	31.31
PAG 20 (NP_788802.1)	100	0.27	1	1	969	47.63
PAG 21 (NP_788803.1)	100	0.17	2	3	595	14.47

List of identified boPAGs in the sample from late gestation (for an SDS-PAGE gel image of the samples please see Figure 1a: Lane 5 and 6).

Table S3. List of identified boPAGs in the sample from late gestation (glycosylated).

PAG	Protein Identification Probability [%]	Protein Percentage of Total Spectra [%]	Exclusive Unique Peptide Count	Exclusive Unique Spectra Count	Total Spectrum Count	Percentage of Amino Acids Identified [%]
PAG 1 (NP_776836.1)	100	0.65	18	35	2288	61.32
PAG 3 (NP_001291497.1)	100	0.09	1	2	309	28.08
PAG 4 (NP_788788.1)	100	0.10	4	5	333	12.63
PAG 6 (NP_788790.1)	100	0.08	1	1	294	15.30
PAG 7 (NP_001103448.1)	100	0.22	1	1	759	47.37
PAG 10 (NP_788794.2)	100	0.09	1	2	307	28.87
PAG16 (NP_788798.1)	100	0.20	4	4	713	28.28
PAG 20 (NP_788802.1)	100	0.15	1	1	523	28.42
PAG 21 (NP_788803.1)	100	0.15	1	2	523	8.16

Table S4. List of identified boPAGs in the sample from late gestation (deglycosylated).

PAG	Protein Identification Probability [%]	Protein Percentage of Total Spectra [%]	Exclusive Unique Peptide Count	Exclusive Unique Spectra Count	Total Spectrum Count	Percentage of Amino Acids Identified [%]
PAG 1 (NP_776836.1)	100	0.84	17	31	3016	65.00
PAG 3 (NP_001291497.1)	100	0.13	1	3	472	25.72
PAG 4 (NP_788788.1)	93	0.13	0	0	454	12.63
PAG 6 (NP_788790.1)	100	0.13	3	3	469	26.12
PAG 7 (NP_001103448.1)	100	0.25	1	1	915	35.79
PAG 10 (NP_788794.2)	100	0.09	1	1	322	24.93
PAG16 (NP_788798.1)	100	0.24	3	4	849	22.98
PAG 20 (NP_788802.1)	100	0.21	1	1	741	28.42
PAG 21 (NP_788803.1)	100	0.15	1	2	546	8.42

List of identified boPAGs in the sample from afterbirth (for an SDS-PAGE gel image of the samples please see Figure 1a: Lane 7 and 8).

Table S5. List of identified boPAGs in the sample from afterbirth (glycosylated).

PAG	Protein Identification Probability [%]	Protein Percentage of Total Spectra [%]	Exclusive Unique Peptide Count	Exclusive Unique Spectra Count	Total Spectrum Count	Percentage of Amino Acids Identified [%]
PAG 1 (NP_776836.1)	100	0.61	16	27	2207	58.68
PAG 3 (NP_001291497.1)	100	0.10	1	1	351	28.61
PAG 4 (NP_788788.1)	100	0.12	13	18	439	54.74
PAG 6 (NP_788790.1)	100	0.10	2	2	353	21.90
PAG 7 (NP_001103448.1)	100	0.24	1	1	860	34.47
PAG 10 (NP_788794.2)	100	0.19	2	3	668	55.91
PAG16 (NP_788798.1)	100	0.24	4	5	871	36.11
PAG 20 (NP_788802.1)	100	0.22	2	2	776	56.84
PAG 21 (NP_788803.1)	100	0.17	2	5	620	15.00

Table S6. List of identified boPAGs in the sample from afterbirth (deglycosylated).

PAG	Protein Identification Probability [%]	Protein Percentage of Total Spectra [%]	Exclusive Unique Peptide Count	Exclusive Unique Spectra Count	Total Spectrum Count	Percentage of Amino Acids Identified [%]
PAG 1 (NP_776836.1)	100	0.79	16	24	2889	66.32
PAG 3 (NP_001291497.1)	100	0.16	1	2	583	28.61
PAG 4 (NP_788788.1)	100	0.17	1	1	623	54.74
PAG 6 (NP_788790.1)	100	0.14	3	3	507	22.16
PAG 7 (NP_001103448.1)	100	0.29	1	1	1046	32.11
PAG 10 (NP_788794.2)	100	0.25	2	3	918	59.06
PAG16 (NP_788798.1)	100	0.29	4	6	1050	36.11
PAG 20 (NP_788802.1)	100	0.31	2	2	1117	57.89
PAG 21 (NP_788803.1)	100	0.17	1	2	604	10.79

List of identified boPAGs in the sample from late gestation (for an SDS-PAGE gel image of the samples please see Figure 1a: Lane 9 and 10).

Table S7. List of identified boPAGs in the sample from late gestation (glycosylated).

PAG	Protein Identification Probability [%]	Protein Percentage of Total Spectra [%]	Exclusive Unique Peptide Count	Exclusive Unique Spectra Count	Total Spectrum Count	Percentage of Amino Acids Identified [%]
PAG 1 (NP_776836.1)	100	0.62	18	35	2210	61.32
PAG 3 (NP_001291497.1)	100	0.09	1	3	304	28.61
PAG 4 (NP_788788.1)	100	0.11	15	19	405	55.00
PAG 6 (NP_788790.1)	100	0.10	1	1	340	19.53
PAG 7 (NP_001103448.1)	100	0.20	1	1	702	50.53
PAG 10 (NP_788794.2)	100	0.09	1	2	303	30.97
PAG16 (NP_788798.1)	100	0.19	6	8	666	45.20
PAG 20 (NP_788802.1)	100	0.26	5	9	919	61.84
PAG 21 (NP_788803.1)	100	0.13	1	4	444	13.16

Table S8. List of identified boPAGs in the sample from late gestation (deglycosylated).

PAG	Protein Identification Probability [%]	Protein Percentage of Total Spectra [%]	Exclusive Unique Peptide Count	Exclusive Unique Spectra Count	Total Spectrum Count	Percentage of Amino Acids Identified [%]
PAG 1 (NP_776836.1)	100	0.86	21	36	2896	82.11
PAG 3 (NP_001291497.1)	100	0.13	1	3	426	31.23
PAG 4 (NP_788788.1)	100	0.14	1	1	470	53.95
PAG 6 (NP_788790.1)	100	0.15	4	5	497	27.44
PAG 7 (NP_001103448.1)	100	0.28	1	1	935	38.68
PAG 10 (NP_788794.2)	100	0.09	1	2	311	35.43
PAG16 (NP_788798.1)	100	0.28	6	7	934	44.70
PAG 20 (NP_788802.1)	100	0.39	3	5	1304	60.53
PAG 21 (NP_788803.1)	100	0.16	1	2	548	11.05

List of identified boPAGs in the sample from late gestation (for an SDS-PAGE gel image of the samples please see Figure 1b: Lane 3 and 4).

Table S9. List of identified boPAGs in the sample from late gestation (glycosylated).

PAG	Protein Identification Probability [%]	Protein Percentage of Total Spectra [%]	Exclusive Unique Peptide Count	Exclusive Unique Spectra Count	Total Spectrum Count	Percentage of Amino Acids Identified [%]
PAG 1 (NP_776836.1)	100	0.49	18	30	1731	60.53
PAG 3 (NP_001291497.1)	100	0.07	1	2	246	24.93
PAG 4 (NP_788788.1)	100	0.07	4	5	232	12.89
PAG 6 (NP_788790.1)	100	0.07	4	5	259	25.59
PAG 7 (NP_001103448.1)	100	0.15	1	1	527	45.26
PAG 10 (NP_788794.2)	100	0.05	0	0	171	12.34
PAG16 (NP_788798.1)	100	0.14	3	3	493	20.20
PAG 20 (NP_788802.1)	100	0.12	1	1	416	26.58
PAG 21 (NP_788803.1)	100	0.11	1	4	381	8.16

Table S10. List of identified boPAGs in the sample from late gestation (deglycosylated).

PAG	Protein Identification Probability [%]	Protein Percentage of Total Spectra [%]	Exclusive Unique Peptide Count	Exclusive Unique Spectra Count	Total Spectrum Count	Percentage of Amino Acids Identified [%]
PAG 1 (NP_776836.1)	100	0.69	18	29	2181	66.05
PAG 3 (NP_001291497.1)	98	0.11	1	2	358	20.73
PAG 4 (NP_788788.1)	87	0.08	0	0	261	12.63
PAG 6 (NP_788790.1)	100	0.11	3	3	359	26.39
PAG 7 (NP_001103448.1)	100	0.20	1	1	626	35.26
PAG 10 (NP_788794.2)	95	0.06	0	0	179	13.65
PAG16 (NP_788798.1)	100	0.19	2	2	612	16.92
PAG 20 (NP_788802.1)	100	0.16	1	1	499	28.42
PAG 21 (NP_788803.1)	100	0.14	1	2	437	8.16

List of identified boPAGs in the sample from early gestation (for an SDS-PAGE gel image of the samples please see Figure 1b: Lane 5 and 6).

Table S11. List of identified boPAGs in the sample from early gestation (glycosylated).

PAG	Protein Identification Probability [%]	Protein Percentage of Total Spectra [%]	Exclusive Unique Peptide Count	Exclusive Unique Spectra Count	Total Spectrum Count	Percentage of Amino Acids Identified [%]
PAG 1 (NP_776836.1)	100	0.51	19	32	1699	59.21
PAG 3 (NP_001291497.1)	100	0.07	1	3	246	27.03
PAG 4 (NP_788788.1)	100	0.08	3	4	266	12.63
PAG 6 (NP_788790.1)	100	0.11	10	14	373	52.24
PAG 7 (NP_001103448.1)	100	0.20	1	1	669	46.32
PAG 10 (NP_788794.2)	100	0.06	0	0	189	12.34
PAG16 (NP_788798.1)	100	0.16	2	2	522	18.69
PAG 20 (NP_788802.1)	100	0.14	1	1	467	46.58
PAG 21 (NP_788803.1)	100	0.11	1	4	381	10.26

Table S12. List of identified boPAGs in the sample from early gestation (deglycosylated).

PAG	Protein Identification Probability [%]	Protein Percentage of Total Spectra [%]	Exclusive Unique Peptide Count	Exclusive Unique Spectra Count	Total Spectrum Count	Percentage of Amino Acids Identified [%]
PAG 1 (NP_776836.1)	100	0.72	17	27	2602	64.21
PAG 3 (NP_001291497.1)	100	0.12	1	2	432	23.10
PAG 4 (NP_788788.1)	93	0.10	0	0	369	12.63
PAG 6 (NP_788790.1)	100	0.12	3	3	440	26.39
PAG 7 (NP_001103448.1)	100	0.22	1	1	780	36.32
PAG 10 (NP_788794.2)	89	0.07	0	0	247	9.97
PAG16 (NP_788798.1)	100	0.20	2	2	732	19.44
PAG 20 (NP_788802.1)	100	0.19	1	1	676	46.58
PAG 21 (NP_788803.1)	100	0.13	2	3	472	12.37

Table S13. Initial list of proteins and proteotypic peptides.

PAG	Peptide Sequence	Precursor Charge	Precursor *m/z*
PAG 1 (NP_776836.1)	K.ALVDTGTSDIVGPR.R	2	700.87
	R.AISEPVFAFYLSK.D	2	736.39
	R.VSSSTETWYLGDVFLR.L	3	620.64
PAG 2 (NP_788787.1)	R.QYFSVFDR.K	2	531.25
	K.TFNPQNSSSFR.E	2	642.80
	R.NYLDTAYVGNITIGTPPQEFR.V	3	790.39
PAG 3 (NP_001291497.1)	K.VSSSTETWILGDVFLR.V	3	603.98
PAG 4 (NP_788788.1)	K.ALVDTGSSDIVGPSTLVNNIWK.L	3	762.73
PAG 5 (NP_788789.1)	K.TFSITYGSGSTK.G	2	624.80
	R.HLESSTSGLTQK.T	2	644.33
	K.ENTVSTSTETWILGDVFLR.L	3	723.36
PAG 6 (NP_788790.1)	K.ALVDTGTSDIVGPSTLVNNIWK.L	3	767.40
	K.GIPFDGILGLSYPNK.T	3	530.95
	R.HHQSSTFRPTNK.T	2	720.36
PAG 7 (NP_001103448.1)	R.STESWVLGEVFLR.L	2	761.89
	R.HLQSSTFRPTNK.T	3	472.58
	K.WVPLIQAVDWSVHVDR.I	3	640.67
PAG 8 (NP_788792.2)	K.NLGTSETWILGDVFLR.L	3	607.65
PAG 9 (NP_788793.1)	R.QLQSSTFQPTNK.T	2	689.85
	K.GELNWIPLIEAGEWR.V	3	594.97
	K.YLPSITFIINGIK.Y	2	739.93
PAG 10 (NP_788794.2)	K.AANDQNIIYHHPLR.S	3	554.62
	R.IGNLVSVAQPFGLSLK.E	3	548.32
	R.TITGANPIFDNLWK.Q	2	795.41
PAG 11 (NP_788796.1)	K.LLNSFLEEQANR.M	2	717.37
	K.QQGAISEPIFAFYLSTR.K	3	643.33
	R.VVFDTGSSDLWVPSIK.C	3	583.97
PAG 12 (NP_788795.1)	K.GELNWIPVSQTR.Y	2	700.37
PAG 14 (XP_002699292.1)	R.NISFSGAIPIFYK.L	2	728.89
	R.DLFYVGNITIGTPPQK.F	3	588.31
PAG 15 (NP_788797.1)	R.LSQISFHGSNLTIHPLR.N	3	640.68
PAG16 (NP_788798.1)	R.STESWLLGDVFLR.L	2	761.89
	R.HFQSSTFRPTTK.T	2	718.86
	K.NQGAISDPIFAFYLSK.D	3	590.97
PAG 17 (NP_788800.1)	K.EHTYSLSQISSR.G	3	469.90
	K.GELNWVPLIQAGGWTVHVDR.I	3	749.72
PAG 18 (NP_788799.1)	K.AVVDTGTSLIEGPR.R	3	472.25
	R.LSPPSTETWILGDVFLR.R	3	644.34
	K.LSFSGAIPIFDNLR.N	3	517.28
PAG 20 (NP_788802.1)	R.FDGVLGLNYPNISFSK.A	3	590.97
	R.STEFWILGEAFLR.L	3	523.60
PAG 21 (NP_788803.1)	R.QHQSSTFRPTNK.T	2	715.86
	R.IGDLVSTDQPFGLSVSEYGFK.D	3	753.71
	K.NEGAISEPIFAFYLSK.K	3	595.97
